# The Extraordinary Evolutionary History of the Reticuloendotheliosis Viruses

**DOI:** 10.1371/journal.pbio.1001642

**Published:** 2013-08-27

**Authors:** Anna Maria Niewiadomska, Robert J. Gifford

**Affiliations:** Aaron Diamond AIDS Research Center, New York, New York, United States of America; University of Wisconsin-Madison, United States of America

## Abstract

The reticuloendotheliosis viruses (REVs) comprise several closely related amphotropic retroviruses isolated from birds. These viruses exhibit several highly unusual characteristics that have not so far been adequately explained, including their extremely close relationship to mammalian retroviruses, and their presence as endogenous sequences within the genomes of certain large DNA viruses. We present evidence for an iatrogenic origin of REVs that accounts for these phenomena. Firstly, we identify endogenous retroviral fossils in mammalian genomes that share a unique recombinant structure with REVs—unequivocally demonstrating that REVs derive directly from mammalian retroviruses. Secondly, through sequencing of archived REV isolates, we confirm that contaminated *Plasmodium lophurae* stocks have been the source of multiple REV outbreaks in experimentally infected birds. Finally, we show that both phylogenetic and historical evidence support a scenario wherein REVs originated as mammalian retroviruses that were accidentally introduced into avian hosts in the late 1930s, during experimental studies of *P. lophurae*, and subsequently integrated into the fowlpox virus (FWPV) and gallid herpesvirus type 2 (GHV-2) genomes, generating recombinant DNA viruses that now circulate in wild birds and poultry. Our findings provide a novel perspective on the origin and evolution of REV, and indicate that horizontal gene transfer between virus families can expand the impact of iatrogenic transmission events.

## Abstract 2

Reticuloendotheliosis viruses are mammalian retroviruses that were transmitted to avian hosts through inadvertent human intervention, and subsequently integrated their genetic material into the genomes of large DNA viruses, generating novel recombinant pathogens that now circulate naturally in poultry and wild birds.

## Author Summary

Retroviruses are characterized by their ability to insert a DNA copy of their genome into the chromosomes of infected cells. Occasionally, retroviruses insert into “germline” cells and are subsequently inherited as host alleles called endogenous retroviruses (ERVs). Vertebrate genomes contain thousands of ERV sequences derived from ancient retroviruses, and these viral sequences serve as molecular “fossils” that can be used to explore how retroviruses have evolved over millions of years. Here we combine an analysis of the retroviral “fossil record” with a phylogenetic and historical investigation to determine the origin of a group of avian retroviruses called reticuloendotheliosis viruses (REVs). We present evidence to demonstrate that rather than arising from natural infections of birds, REVs are in fact derived from mammalian retroviruses that were accidentally introduced into avian hosts during experimental studies of a malaria parasite in the late 1930s. Remarkably, REVs have subsequently inserted into the genomes of two large DNA viruses that infect birds, generating chimeric viruses that now circulate naturally in poultry and wild birds.

## Introduction

The reticuloendotheliosis viruses (REVs) comprise several closely related amphotropic retroviruses (family *Retroviridae*) isolated from birds [1]. The prototypic REV isolate was isolated from a turkey in 1957 [2]. Subsequently, REV infections have been reported in a diverse range of gamebirds (order Galliformes) and waterfowl (order Anseriformes). Infection is associated with a range of disease syndromes, including anemia, immunosuppression, neoplasia, runting, and feathering abnormalities called “nakanuke.” The etiology of REV infection remains enigmatic—although antibodies to REV are widespread in poultry, REV outbreaks occur only sporadically and are relatively rare [3].

All retroviruses replicate their genomes via a DNA intermediate that is integrated into the nuclear DNA of the host cell and is referred to as a “provirus.” Occasionally, infection of germ cells allows retroviral proviruses to enter the host germline, so that they can be vertically inherited as host alleles, called *endogenous retroviruses* (ERVs) [4], a proportion of which end up becoming fixed in the germline. These ancestral retrovirus sequences represent retroviral “fossils” [5],[6], and as such they support “paleovirological” investigations that seek to address the long-term, macroevolutionary history of interaction between hosts and retroviruses [7],[8]. In a previous study, phylogenetic analysis of retroviral polymerase (*pol*) gene sequences revealed that REV groups robustly within the Gammaretrovirus genus, and is closely related to an ERV in the genome of the short-beaked echidna (*Tachyglossus aculeatus*)—an-egg laying mammal found only in Australia and New Guinea [9]. This discovery reinforced the conclusions of earlier, serological studies, which proposed REVs to have originated in mammals [10].

Curiously, sequences derived from REV have also been identified in the genomes of two large DNA viruses that naturally infect birds: fowlpox virus (FWPV), a poxvirus [11],[12] (family *Poxviridae*), and gallid herpesvirus 2 (GHV-2), a herpesvirus (family *Herpesviridae*). FWPV infects poultry and wild birds throughout the world, and causes a mild-to-severe, slow developing disease (avian pox) characterized by the formation of proliferative external lesions (dry pox), and diphtheritic lesions in the digestive and respiratory tracts (wet pox) [13]. GHV-2 is the causative agent of Marek's disease, a highly contagious disease of chickens and other galliform birds that is associated with a wide range of clinical syndromes, including neoplasia and paralysis [14]. Clinical disease is not always apparent in infected birds, but mortality rates in susceptible flocks can be very high [14].

Contamination of both FWPV and Marek's disease vaccines with replication competent REV, leading to outbreaks of REV infection, has been reported on numerous distinct occasions [3],[15]. However, only remnant REV sequences, incapable of expressing retrovirus, have been identified in GHV-2 and FWPV vaccine strains (typically a “solo LTR” derived from the long terminal repeat (LTR) regions that flank the provirus) [12],[16]. By contrast, FWPV field strains containing near full-length REV proviruses appear to circulate naturally in unvaccinated birds [12],[16]–[19]. Recently, a field strain of GHV-2 containing a novel REV LTR insertion was reported [20].

In this study, we used a combination of PCR-based and *in silico* screening to explore the origin and evolutionary history of the REV lineage, and to investigate the processes linking exogenous REV isolates with endogenous REV-related sequences in virus and animal genomes.

## Results

### Paleovirological History of the REV Lineage

**Figure 1:**
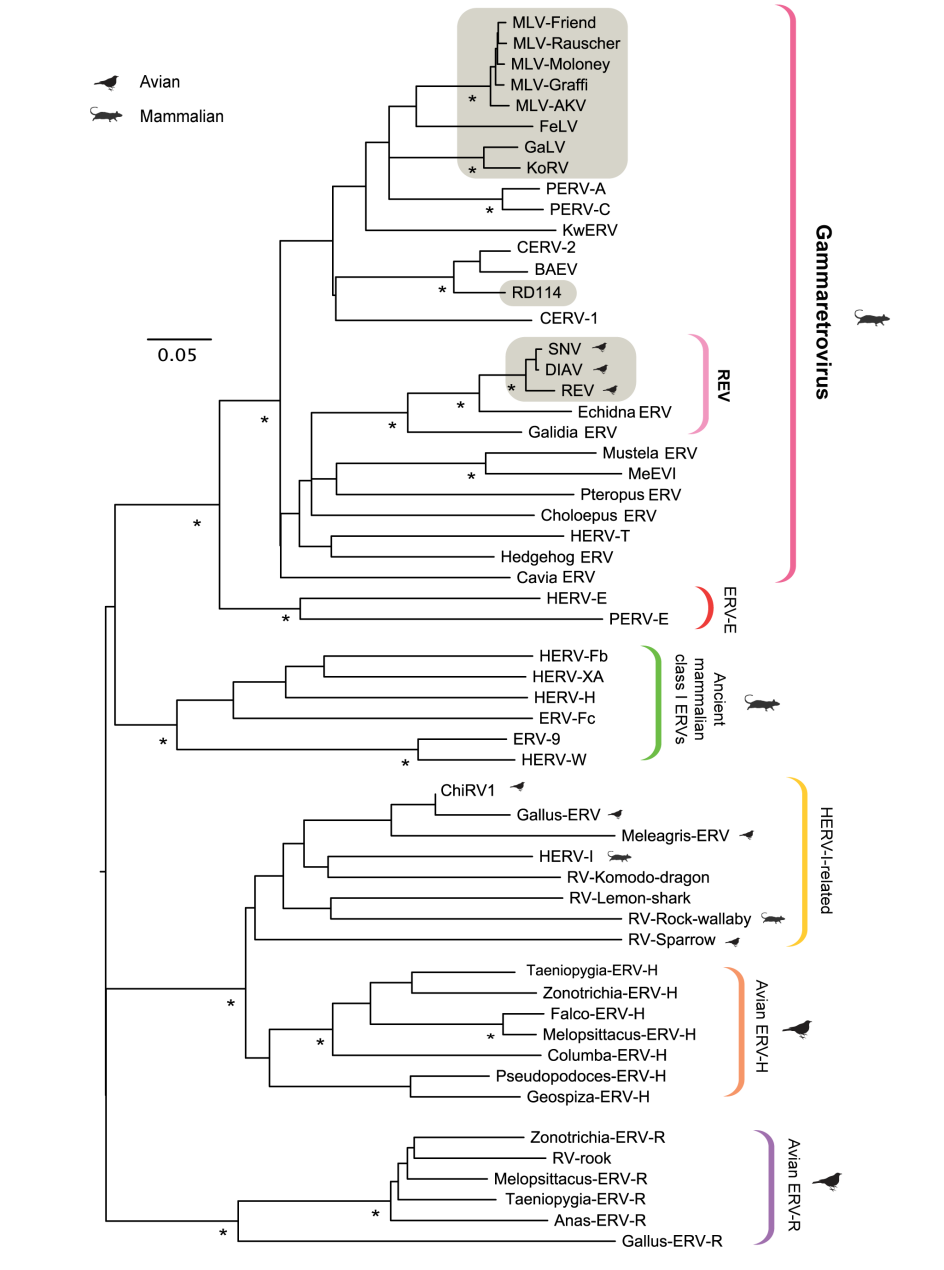
Evolutionary relationships among the RT genes of exogenous Gammaretroviruses and related ERVs. Shaded boxes indicate taxa that are known to occur as exogenous retroviruses. Brackets to the right indicate major lineages (note: an integrated taxonomy of exogenous and ERVs has yet to be established by the International Committee on Taxonomy of Viruses, and the groupings shown here are propositional). Associations of retrovirus groups and individual retroviral taxa with avian and mammalian hosts are indicated, as shown in the key. The phylogeny shown was constructed using NJ and a multiple sequence alignment spanning 140 amino acid residues in the reverse transcriptase protein (RT), and is midpoint rooted for display purposes. To obtain putative protein sequences for ERVs, frameshifting indels were inferred and removed, and the resulting nucleotide sequence was conceptually translated. Asterisks indicate clades with bootstrap support >90% in both NJ and maximum likelihood (ML) trees, based on 1,000 bootstrap replicates. The scale bar indicates evolutionary distance in substitutions per site. Table S2 provides details of all the ERVs and exogenous retrovirus taxa shown in the phylogeny.

To investigate the deeper origins of the REVs, we screened avian and mammalian genome sequence databases (Table S1) for ERV sequences closely related to REV (Table 1). Screening of 42 mammalian genomes identified numerous ERV loci that disclosed highly significant similarity to one or more REV coding domains, but none that matched closely to REV across the entire coding region of the genome. We found that all mammalian ERVs exhibiting a high degree of sequence similarity to REV in the *gag*-*pol* domain exhibited no such similarity in *env*, and vice versa. This can be assumed to reflect the recombinant genome structure of REV [21],[22], comprising a Gammaretrovirus *gag-pol* domain fused to an *env* domain that is more commonly associated with the Betaretrovirus genus (although it also occurs in some other Gammaretroviruses, also considered to be recombinants [23]). No ERV loci closely related to REV were detected in avian genomes. We did identify numerous avian ERVs that disclosed weak similarity to REV in *pol* (30–40% amino acid identity). However, phylogenetic analysis revealed these ERVs to be derived from ancient, highly degenerated ERV lineages that were clearly distinct from modern Gammaretroviruses (Figure 1).

**Table 1:** Distribution of REV-related sequences in vertebrate and virus genomes.

		No. of Highly Significant Matches Detected^b^			
Species/Group^a^	LTR	Leader	Gag	Pol	Env
**Mammals**					
European hedgehog (*Erinaceus europaeus*)	—	—	76	166	—
Madagascar tenrec (*Echinops telfairi*)	—	—	—	5	—
Flying fox (*Pteropus vampyrus*)	—	—	7	14	—
Little brown bat (*Myotis lucifugus*)	—	—	9	3	—
Ferret (*Mustela putroius furo*)	—	—	—	4	—
Hoffmann's two toed sloth (*Choloepus hoffmanni*)	—	—	—	7	—
Gray short-tailed opossum (*Monodelphis domestica*)	—	—	—	4	—
Nine-banded armadillo (*Dasypus novemcinctus*)	—	—	—	1	—
Phillipine tarsier (*Tarsius syrichta*)	—	—	—	—	3
Cape hyrax (*Procavia capensis*)	—	—	1	—	13
**Large DNA viruses**					
Marek's disease virus (MDV)	4	—	—	—	—
Fowlpox virus (FWPV)	15	2	2	2	1

a Only species in which highly significant matches were detected are shown. Table S2 provides a complete list of avian and mammalian species for which whole genome sequence data were screened. For large DNA viruses we screened all “*Herpesviridae*” and “*Poxviridae*” sequences in GenBank.

b Highly significant matches were identified using an empirically determined BLAST bitscore threshold (see Materials and Methods).

**Figure 2:**
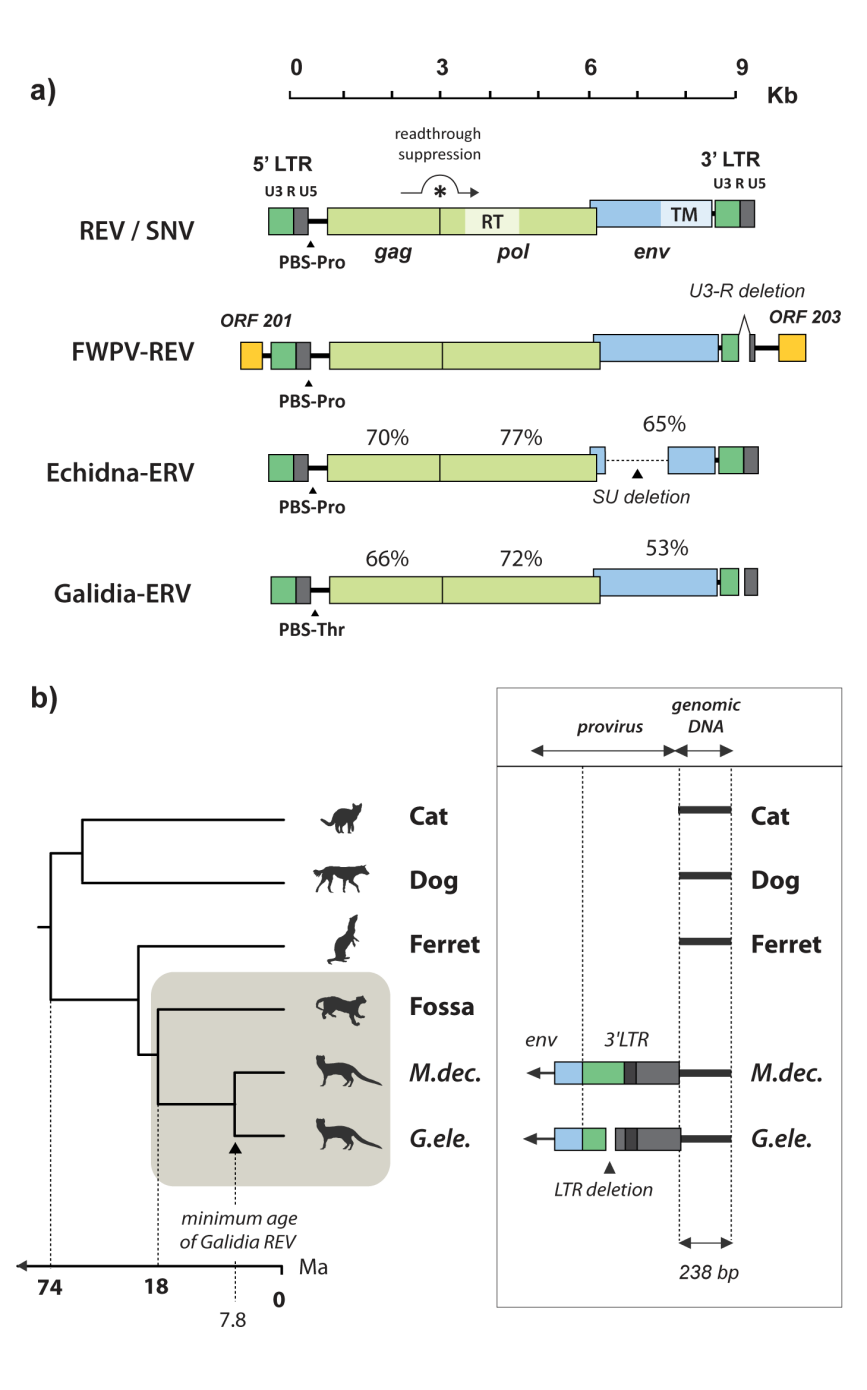
Genomic and paleovirological characteristics of REV-related retroviruses. The schematic in panel (a) shows the genome structure of REV and SNV, a near full-length REV insertion in the FWPV genome, and the mammalian ERVs Echidna-ERV and *Galidia*-ERV. Percentage sequence identity to SNV, at the amino acid level, is shown for the putative Gag, Pol, and Env polyproteins of Echidna-ERV and *Galidia*-ERV. Proviral coding regions that disclose homology to Gammaretroviruses are shown in green, whereas those that disclose homology to Betaretroviruses are shown in blue. ORFs flanking the REV insertion in FWPV are in yellow. Panel (b) summarizes the genomic data used to estimate the minimum age of REV-related ERV insertions in Malagasy carnivore genomes. A time-scaled Carnivora phylogeny (based on Nyakatura et al. [27]) is shown on the left, with Malagasy carnivores shaded. A corresponding schematic on the right shows the genomic locus at which an orthologous ERV insertion was identified in a subset of Malagasy carnivores. Boxes represent the *env* gene (blue) and 3′ LTR sequences (green = U3; dark grey = R; light grey = U5). The adjacent black line represents flanking genomic DNA, spanning 238 nucleotides, obtained from the striped mongoose (*Mungotictis decemlineata*) and ring-tailed mongoose (*Galidia elegans*) genomes in our study, and aligned to a homologous genomic region (lacking a proviral insertion) in the cat (*Felis catus*), dog (*Canis familiaris*), and ferret (*Mustela furo*) genomes. An orthologous ERV insertion was detected in *M. decemlineata* and *G. elegans* genomes, but not in the more distantly related Fossa (*Cryptoprocta ferox*), indicating that germline invasion occurred between 18 and 8 Ma. Genetic data indicate that all Malagasy carnivores are derived from a single founder population that colonized Madagascar ∼19 Ma [26]; thus, invasion of the Malagasy carnivore germline occurred in Madagascar. The nucleotide sequence alignment on which the schematic in panel (b) is based on is shown in Figure S1. Abbreviations: RV, retrovirus; Kb, Kilobases; ORF, open reading frame; PBS, primer binding site; Pro, proline; Thr, threonine; LTR, long terminal repeat; U3, unique three prime region; R, repeat region; U5, Unique five prime region; RT, reverse transcriptase; SU, surface protein; TM, transmembrane protein; M.dec, *Mungotictis decemlineata*; G.ele, *Galidia elegans*.

**Figure 3:**
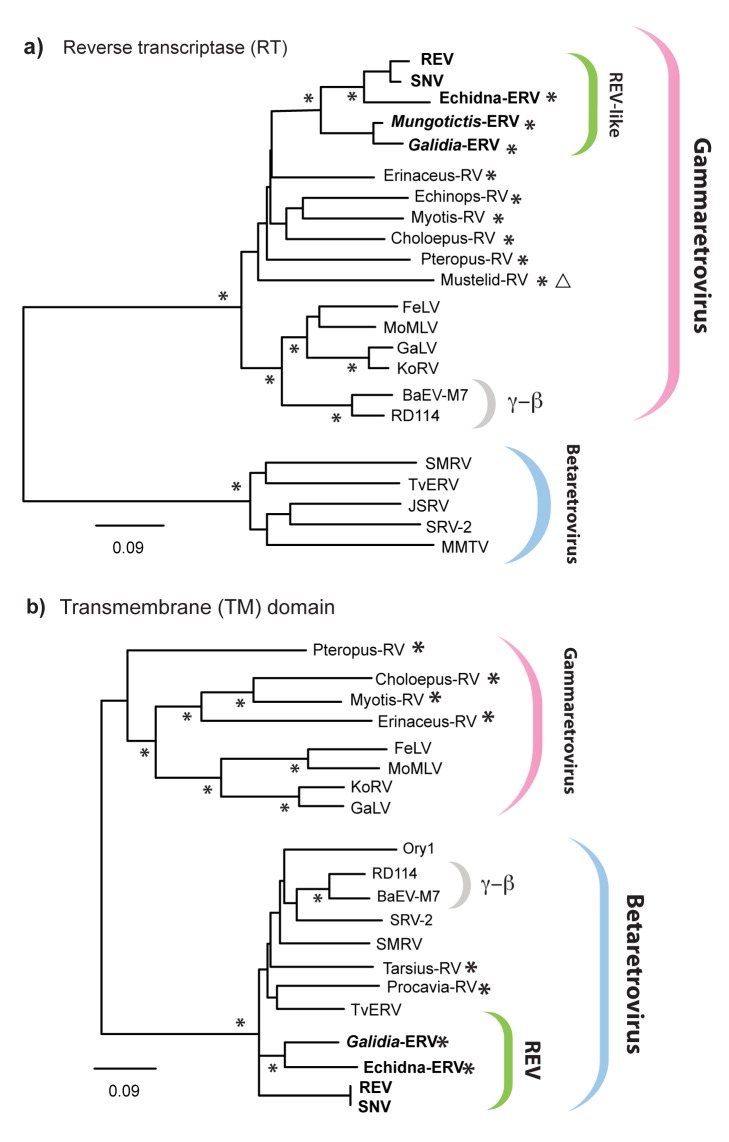
Contrasting phylogenetic relationships of *pol* and *env* genes found in REV-related retroviruses. Panels (a) and (b) show ML phylogenies constructed from alignments of Gamma- and Betaretrovirus protein sequences. The phylogeny in panel (a) was constructed from an alignment spanning 157 residues of the RT protein encoded by *pol*, whereas the phylogeny in panel (b) was constructed from an alignment spanning 153 residues of the TM domain in the polypeptide encoded by *env*. Asterisks on internal nodes indicate ML bootstrap support >95%, (based on 1,000 bootstrap replicates). Asterisks beside taxa names indicate ERV families identified in this study. Open triangles indicate ERV lineages for which *env* genes were not identified. Scale bars indicate evolutionary distance in substitutions per site. Brackets to the right indicate genus designations, and viruses previously identified as Gamma- and Betaretrovirus (γ-β) recombinants. Table S2 provides details of all the ERVs and exogenous retrovirus taxa shown in the phylogeny. Abbreviations: RV, retrovirus; MoMLV, Moloney murine leukemia virus; FeLV, feline leukemia virus; GaLV, gibbon ape leukemia virus; KoRV, koala retrovirus; BAEV, baboon endogenous virus; SMRV, squirrel monkey retrovirus; TvERV, *Trichosurus vulpecula* endogenous retrovirus; JSRV, Jaagsiekte sheep retrovirus; SRV, simian retrovirus; MMTV, mouse mammary tumor virus.

In phylogenies based on reverse transcriptase (RT), avian REV isolates cluster tightly with a previously described ERV sequence derived from the short-beaked echidna genome [9]. During a polymerase chain reaction (PCR)–based investigation of ERV diversity in Malagasy mammals, we serendipitously identified additional ERV RT sequences that grouped within this clade, in the genomes of two Malagasy carnivore species: the ring-tailed mongoose (*Galidia elegans*) and the narrow-striped mongoose (*Mungotictis decemlineata*). We recovered near complete proviral genome sequences for all three REV-related ERVs (hereafter referred to as echidna-ERV, *Galidia*-ERV, and *Mungotictis*-ERV) (Figure 2a), revealing that they exhibit similarity to REV throughout the entire internal coding region of the genome. Crucially, echidna-ERV, *Galidia*-ERV, and *Mungotictis*-ERV grouped robustly with REV isolates in phylogenies constructed using both the *pol* and *env* coding domains (Figure 3a and 3b), establishing that they share a common, recombinant ancestor with these viruses. Thus, ERVs belonging to the REV-lineage do occur in the genomic fossil record of mammals, but as with certain other retrovirus groups, such as foamy viruses and lentiviruses, they are relatively rare [24],[25].

PCR results suggested that all three ERVs are low copy number (1–2 proviruses) in their host species. Along with other factors, such as the relatively short length of LTRs, this precluded the confident use of molecular clock-based approaches to date the echidna-ERV, *Galidia*-ERV, and *Mungotictis*-ERV insertions. Notably, however, internal coding regions in all three ERVs were relatively intact (although echidna-ERV has a large deletion in region of the *env* gene encoding the surface (SU) glycoprotein (Figure 2a)).

Using a ligation-mediated PCR method, we recovered matching flanking insertion sites for *Galidia*-ERV and *Mungotictis*-ERV, confirming that REV-like viruses occur as orthologous insertions in distinct Malagasy mongoose species. This finding indicates that REV-like viruses entered the germline Malagasy mammals prior to the divergence of *Galidia* and *Mungotictis* ∼8 million years ago (Ma) [26],[27] (Figure 2b). REV-related ERVs were not detected in the more distantly related fossa (*Cryptoprocta ferox*). Together these findings establish that the entire REV lineage—including both mammalian and avian isolates—derives from a common founder that was generated by recombination, and circulated among mammals during the Miocene Epoch (∼23–5 Ma).

### Origin of the Avian REVs

**Figure 4:**
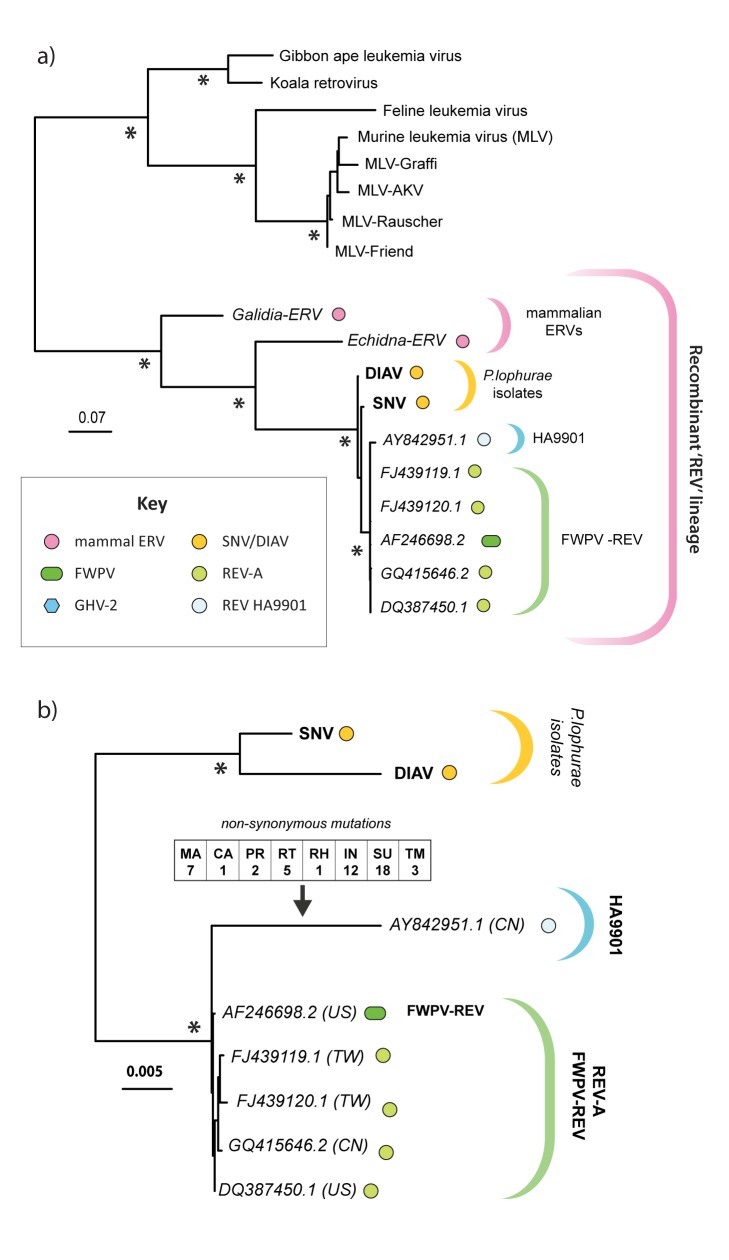
Phylogenetic relationships of REV coding regions. ML phylogenies constructed using (a) an alignment spanning residues 183–481 of the Pol polyprotein (DIAV coordinates) and containing REV and mammalian gammaretroviruses sequences and (b) a nucleotide alignment of the entire internal coding region of full-length avian isolates. The tree in panel (b) indicates the number of strain-specific, nonsynonymous mutations estimated to have occurred in the nucleocapsid (NC), capsid (CA), matrix (MA), protease (PR), RT, RNase-H (RH), integrase (IN), surface (SU), and TM genes of the exogenous isolate HA9901. Asterisks on internal nodes indicate ML bootstrap support >95%. All trees are midpoint rooted for display purposes. Scale bars indicate evolutionary distance in substitutions per site. Taxa labels include sequence accession numbers, and in panel (b) two-letter ISO country codes enclosed by brackets indicating the country of sampling. Further details of REV sequences included in these trees can be found in Table S4.

**Figure 5:**
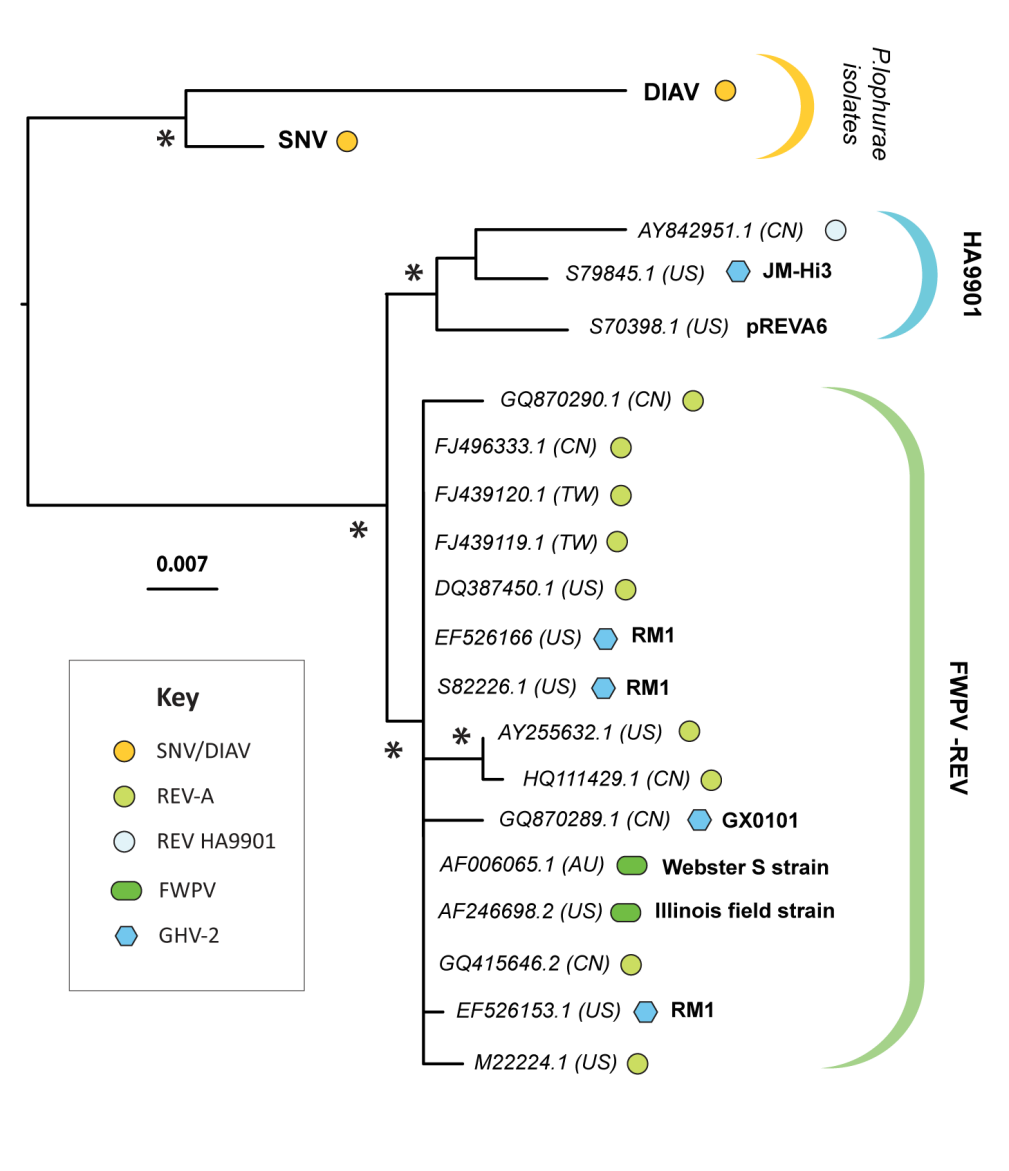
Phylogenetic relationships of REV LTR sequences. ML phylogenies constructed using an alignment of REV LTR sequences. Asterisks on internal nodes indicate ML bootstrap support >95%. The phylogeny is midpoint rooted for display purposes. Scale bars indicate evolutionary distance in substitutions per site. Taxa labels include two-letter ISO country codes indicating the country of sampling.. Taxa labels include accession numbers and two-letter ISO country codes enclosed by brackets indicating country of sampling. Where appropriate, FWPV and GHV-2 strain designations are shown in bold. Further details of REV sequences used in the tree, and an alignment figure highlighting lineage-specific LTR indels, can be found in Table S4 and Figure S2, respectively.

Given that the REV lineage clearly originates in mammals, we decided to investigate the origins of the avian REVs in greater detail. We reviewed all published reports of REV outbreaks, and sought to obtain any available archived samples (Table 2). Among REV isolates that had not previously been sequenced, we were only able to obtain one (duck infectious anemia virus (DIAV)), for which all samples had not been exhausted or destroyed. We sequenced the complete DIAV genome and constructed ML phylogenies using all available REV sequence data. Phylogenies were constructed using alignment partitions representing (i) a conserved region of the *pol* coding domain (Figure 4a), (ii) the complete internal coding region of the viral genome (Figure 4b), and (iii) LTR sequences (Figure 5).

**Table 2:** REV publication timeline.

Number	Virus	Secondary Association	Host Species	Host Animal Status	Year	Country	Method(s) of REV Detection	Ref.
1*	*NK*	*P. lophurae*	Duck	Experimental	1941	US	F	[49]
2*	*NK*	*P. lophurae*	Duck	Experimental	1945	US	F	[30]
3†	*FWPV-REV*				1949	US	E	[52]
4†	**SNV**	*P. lophurae*	Duck	Experimental	1958	US	A	[28]
5	**REV**		Turkey	Domestic	1958	US	A	[2]
6	*NK*	*P. lophurae*	Duck	Experimental	1966	US	F	[66]
7†	**CSV**		Chicken		1969	US	A	[67]
8	**DIAV**	*P. lophurae*	Duck	Experimental	1971	US	A, C, D	[32]
9	**REV**		Japanese quail	Domestic	1972	CA	D	[68]
10†	**REV**		Duck	Wild	1973	AU	C, D	[69]
11	**REV**		Muscovy duck	Domestic	1974	US	C, D	[70]
12†	**REV**		Japanese quail	Domestic	1974	MX	C, D	[71]
13	**REV**		Turkey	Domestic	1974	US	A, D	[72]
14	**REV**	GHV-2 vaccine	Chicken	Domestic	1974	JP	A, B, C, D	[15]
15	**REV**	HVT vaccine	Turkey	Domestic	1975	JP	A	[73]
16	**REV**		Turkey	Domestic	1975	UK	A, C, D	[74]
17	**REV**		Turkey	Domestic	1976	US	C, D	[75]
18	**REV**	FWPV	Turkey	Domestic	1976	CA	B, C	[34]
19†	**REV**		Chicken	Domestic	1978	AU	C, D	[76]
20	**SNV (RU1)**	*P. lophurae*	Duck	Experimental	1983	US	A, C, D	[31]
21	**REV**	FWPV	Pheasant	Domestic	1983	HU	A, B, C, D	[35]
22	**REV**		Chicken	Domestic	1984	US	A, C	[77]
23	**REV**		Turkey	Domestic	1988	HU	F	[78]
24	**REV**		Goose	Domestic	1989	HU	A, C, D	[79]
25†	**REV**		Turkey	Wild	1989	US	D	[80]
26†	**REV**		Turkey	Wild	1992	US	A, C	[81]
27†	*GHV-2-REV*	GHV-2 vaccine			1993	US	E	[54]
28†	**REV**	FWPV vaccine	Chickens	Domestic	1996	US	C, E	[82]
29	**REV**	FWPV vaccine	Chickens	Domestic	1997	AU	E	[12]
30	**REV**		Indian peafowl	Domestic	1998	US	F	[83]
31†	**REV**	FWPV	Various	Wild/Domestic	1998	AU	A, E	[17]
32†	**REV**	FWPV	Prairie chickens	Domestic	1998	US	C, D, E	[37]
33	*GHV-2-REV*		Turkey, chicken	Domestic	1999	US	E	[84]
34	*FWPV-REV*	FWPV vaccine			2000	US	E	[18]
35	**REV**		Wild turkeys	Wild	2002	US	E	[85]
36†	**REV**		Turkey	Domestic	2002	US	C, E	[86]
37†	**REV**		Partridge	Domestic	2002	US	B, C	[38]
38†	**REV**		Chickens		2003	US	E	[87]
39†	*FWPV-REV*		Various		2003	US	E	[16]
40	**REV**		Chickens	Domestic	2006	CN	A, E	[39]
41	**REV**	HVT, GHV-2, FWPV vaccines	Chickens	Domestic	2006	TW	C, E	[88]
42	**REV**		Prairie chicken	Captive	2006	US	A, E	[89]
43	**REV**	FWPV	Turkey, chicken	Domestic	2006	HR	E	[90]
44	**REV**	FWPV vaccines	Chickens	Domestic	2006	CN	E	[91]
45†	*FWPV-REV*		Chickens	Domestic	2007	US	E	[92]
46	**REV**		Chinese partridge	Domestic	2007	CN	A, C, D, E	[93]
47	**REV**	FWPV, HVT vaccines		Domestic	2009	CN	E	[94]
48	**REV**		Goose	Domestic	2009	CN	A, D, E	[95]
49	**REV**	FWPV vaccine			2010	EG	D, E	[96]
50†	*FWPV-REV*		Chickens	Domestic	2010	GD	A, E	[97]
51†	*FWPV-REV*		Chickens	Domestic	2010	AU	A, B, D, E	[98]
52	*FWPV-REV*		Chickens	Domestic	2011	IN	D, E	[19]

A comprehensive list of published studies in which REV has been detected, isolated, or otherwise implicated, either as an exogenous retrovirus or as an endogenous insert in the FWPV or GHV-2 genomes. Numbers in the first column cross-reference to Figure 7. The primary virus implicated, isolated, or detected in each published report is shown. Exogenous REV strains are shown in bold, and DNA viruses with REV insertions are shown in italics. Where primary virus occurred in the context of contaminated vaccine or with another infectious agent, details of these secondary associations are shown. Infected host species are indicated, along with the status of the animal (domestic/experimental/captive/wild). The country and year in which the primary virus was implicated, isolated, or detected are shown (countries are shown as two-letter ISO country codes). Where reported/applicable, methods of REV detection used in each report are provided as follows: A, isolation & passage; B, electron microscopy; C, serology; D, histopathology; E, PCR and/or sequencing; F, disease pathology (where no other methods used). Serological surveys of REV antigens (see Table S5) are not included here.

* Reports in which presence of REV was not confirmed.

† Studies for which we have confirmed all samples have been exhausted or destroyed.

Abbreviations: SNV, spleen necrosis virus; DIAV, duck infectious anemia virus; FWPV-REV, Fowlpox virus with REV insertion; GHV-2-REV, gallid herpesvirus type 2 with REV LTR insertion; HVT, herpesvirus of turkeys; *N/K*, not known.

All three phylogenies consistently disclosed three major lineages. The first was comprised of spleen necrosis virus (SNV) and DIAV. Both these viruses were isolated from ducks that were experimentally infected with *Plasmodium lophurae* (SNV in 1959 [28] and DIAV in 1972 [29]). The report describing the isolation of DIAV concluded that *P. lophurae* stocks were the source of infection, and demonstrated contamination of stocks in five different laboratories. Sequencing revealed that SNV and DIAV are highly related (∼98% nucleotide identity), despite being isolated 13 years apart, establishing that contaminated stocks have been the source of multiple outbreaks of retroviral infection in *P. lophurae*–infected ducks, dating back as far as 1959, and likely earlier [28]–[32].

A second clade comprised the REV insertion in FWPV and exogenous REV isolates obtained independently in different countries, including the prototypic REV isolates isolated in the United States [2]. In addition, LTR phylogenies revealed this clade to include insertions present in two distinct GHV-2 strains: an attenuated lab strain (RM1 [33]), and a field strain (GX0101 [20]). Virus in this clade exhibit remarkably little genetic variation overall, despite having apparently been maintained in the avian population for at least 50 years [2]. It thus appears unlikely that the exogenous REV isolates in this clade are spreading primarily through horizontal transmission of infectious retrovirus (since this would be expected to generate greater nucleotide sequence diversity among isolates). Instead, phylogenies suggest that exogenous retroviruses are being expressed from a stable FWPV-REV vector that circulates among domestic and wild birds. This would explain why antibodies to REV are widespread in poultry (see Table S5), and why REV infections occur not only in association with contaminated vaccines, but also in wild birds and unvaccinated commercial flocks [3]. Revealingly, several reports describe FWPV or undiagnosed pox-like infections occurring in bird populations shortly prior to the occurrence of REV outbreaks [2],[34]–[38].

The third clade comprised the exogenous REV isolate HA9901, from China [39], as well as LTR sequences obtained from the JM-Hi3 strain of GHV-2, and a REV plasmid (pREVA6 [40]). This clade is robustly supported in bootstrapped phylogenies, and the presence of unique, shared indels in LTRs provides further evidence of common ancestry (Figure S2). These observations establish that HA9901 shares a common history with pREVA6, which can ultimately be traced back to the prototypic REV specimen [2],[41]. Interestingly, HA9901 has acquired numerous nonsynonymous mutations, consistent with ongoing replication as an exogenous retrovirus (Figure 4b).

## Discussion

The data presented in this study unequivocally demonstrate that REVs derive from a retrovirus that circulated in ancestral mammals, and originated through recombination more than 8 Ma. Furthermore, the extremely low genetic diversity observed among all avian REV isolates and sequences indicates a very recent origin for REV in birds (Figure 4 and 5). In previous studies it has generally been assumed that the REVs are a group of *bona fide* avian retroviruses that circulate in wild bird populations. However, phylogenetic evidence indicates that successful transmission of retroviruses, poxviruses, and herpesviruses across host classes is extremely rare, if indeed it occurs at all [9],[42],[43]. While such “long-distance” transmission events, leading to productive virus replication in the new host, likely do occur at an appreciable frequency for these viruses (particularly, for example, when the recipient host is immunocompromised), unless the transmitted virus is able to spread efficiently from host-to-host in the new species, these instances will typically represent evolutionary dead-ends [44].

**Figure 6:**
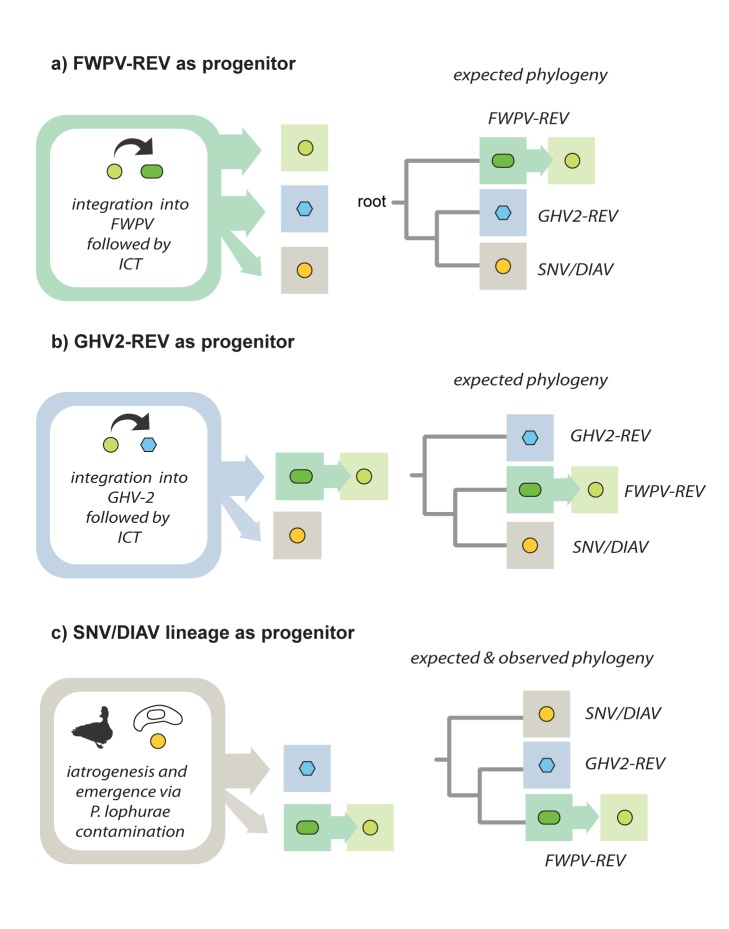
Interclass transmission and the origin of REV. A schematic showing the three possible scenarios via which the ancestor of REV could have crossed from birds (class Aves) into mammals (class Mammalia), assuming a maximum of one *inter-class transmission* (ICT) event in total. For each of the three scenarios shown, the phylogenetic relationships between REV isolates that would be expected to arise as a result are indicated (all phylogenies are rooted on the mammalian ancestor of avian REVs). A REV founder strain could conceivably have been transmitted from mammals to birds after *first* inserting into the genome of FWPV (panel a) or GHV-2 (panel b). However, only a scenario in which the SNV and DIAV lineage were established first (panel c)—as would be expected to occur if *P. lophurae* contamination enabled the iatrogenic emergence of virus—is compatible with the relationships observed in rooted phylogenetic trees (see Figure 4a). Abbreviations: FWPV, fowlpox virus; GHV-2, gallid herpesvirus 2.

**Figure 7:**
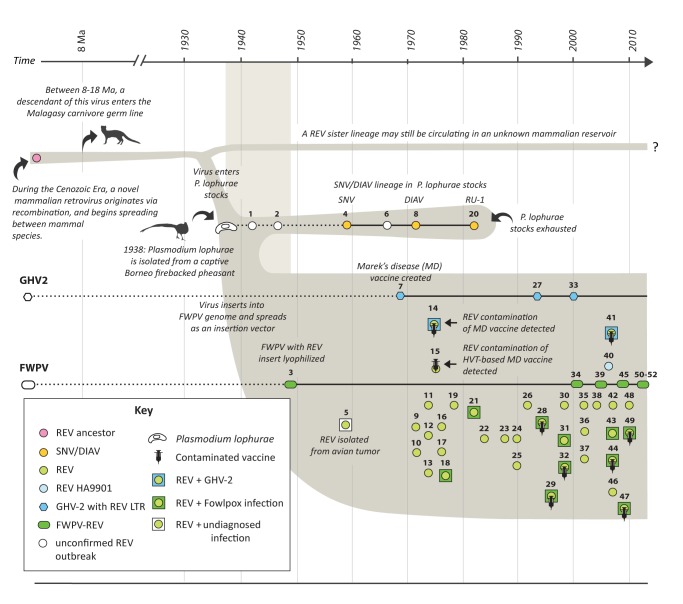
A hypothesis of REV origin and evolution. A schematic representation of REV evolutionary history is shown, summarizing our hypothesis regarding the origin and evolution of the three major avian REV lineages (SNV/DIAV, REV/FWPV-REV, and HA9901) from a mammalian retrovirus ancestor that originated in the Cenozoic Era. REV-associated events (i.e., outbreaks of REV-associated disease, isolation of new REV strains, or identification of REV-containing DNA virus strains) reported in the literature have been mapped onto this schematic, as indicated in the key. Numbers shown above key symbols refer to Table 2, where details of the associated publication or report can be found. The broken scale bar shows time in years A.D. to the right of the break and Ma to the left of the break. A shaded background region indicates the time window for invasion of FWPV genome following iatrogenic introduction into poultry (assuming that reports of REV sequences in FWPV vaccine strains lyophilized in 1949 [52] are accurate). Abbreviations: REV, reticuloendotheliosis virus; SNV, spleen necrosis virus; DIAV, duck infectious anemia virus; FWPV, fowlpox virus; GHV-2, gallid herpesvirus 2; FWPV-REV, Fowlpox virus with REV insertion.

Since REVs clearly originate in mammals, and all avian REVs are highly related, the entire avian REV lineage almost certainly derives from a single founder. Phylogenies rooted on mammalian REVs unambiguously place the SNV/DIAV lineage in a basal position relative to the FWPV-REV and HA9901 clades (Figure 4a). This is most readily reconciled with a scenario wherein REVs originated in *P. lophurae* experiments, and subsequently inserted into the FWPV and GHV-2 genomes (Figure 6). Importantly, this hypothesis of REV origin and evolution is not only consistent with the REV phylogeny, but also with the entire recorded history of REV-associated disease (Figure 7, Table 2), accounting for the disappearance of the SNV/DIAV lineage since the 1980s (when *P. lophurae* stocks were exhausted—see below), and the limited genetic diversity observed among all avian REVs (since relatively few virus replication cycles would be expected to separate all isolates). Moreover, this scenario accounts for the anomaly of retroviral interclass transmission, because it occurs in an experimental context wherein a pathogen (*P. lophurae*) is being deliberately adapted to a foreign host species.

*P. lophurae* has only been isolated once, in June 1937, in the New York Zoological Park (now Bronx Zoo), by Lowell T. Coggeshall. Coggeshall, who was then working for the Rockefeller Foundation, was searching for a parasite that could serve as an experimental model system for malaria research. In 1935, Émile Brumpt of the Pasteur Institute had identified *Plasmodium gallinaceum*, a parasite causing malarial disease in poultry, during an excursion to Ceylon (now Sri Lanka) [45]. However, *P. gallinaceum* could not be introduced to the United States due to strict quarantine regulations against importation of poultry pathogens [46]. Reasoning that other avian species from the same geographic region might harbor a similar parasite, Coggeshall screened some South East Asian bird species that had been introduced to the New York Zoological Park in the 1920s by ornithologist Lee Saunders Crandall [47]. This led to the identification of a plasmodium in the blood of a Borneo firebacked pheasant (*Lophura igniti igniti*), which proved transmissible to very young chickens [48].

Stocks of this parasite, designated *Plasmodium lophurae*, were maintained by serial passage in chicken, duck, and turkey chicks, with 25 passages reported as of 1938 [48]. Published reports suggest that contaminating virus was present from an early stage; a 1941 study of *P. lophurae* noted that anemia in infected animals appeared to be decoupled from parasite replication, indicating the presence of a second infectious agent [49]. A study a few years later confirmed the presence of an additional “filterable agent”—the cause of a lethal anemia—in *P. lophurae*–infected poultry [30], and in 1959 William Trager identified this agent as SNV [28]. Subsequently, SNV-like viruses were isolated from *P. lophurae*–infected ducks on multiple distinct occasions (Table 2) [1],[31. The role of *P. lophurae* stocks as a source of infection appears to have gone unappreciated prior to the isolation of DIAV in 1972 [29]. But while the associated study concluded that “DIAV has been an unrecognized companion of *P. lophurae* for many years,” the assumption remained that the contaminating virus was a natural pathogen of ducks.

Research on *P. lophurae* effectively ceased in the 1980s, when stocks could no longer be replenished. The organism has never subsequently been identified, and thus remains an enigma in many respects. Expeditions to Borneo have been mounted with the express purpose of obtaining further isolates, but these failed to identify the parasite in populations of wild birds [46]. Since *P. lophurae* stocks ran out, no further viruses belonging to the SNV/DIAV lineage have been isolated, consistent with the hypothesis that contaminated stocks were the principle reservoir of infection for these viruses.

It remains unclear whether the progenitors of avian REVs were present in the animal from which *P. lophurae* was originally obtained or were introduced from an external source during serial passage. However, since none of the mammalian species that might be considered likely sources of contamination in a lab environment (i.e., mouse, rat, rabbit, guinea pig) appear to harbor truly REV-like viruses in exogenous or endogenous forms, whereas more exotic mammalian species do, cross-species transmission or contamination within the setting of the zoological park is an attractive hypothesis. Notably, we have identified REV-related ERVs in mammalian groups (Malagasy carnivores and Australian monotremes) that inhabit highly distinct and relatively isolated biogeographic regions, separated from one another by large expanses of ocean. This suggests that infection has been widespread in the past and that chiropteran (bat) vectors were likely at least partly involved in the spread of virus.

It also remains unclear precisely when and how the REV insertions in FWPV and GHV-2 genome were generated. REV could presumably have spread from birds experimentally infected with *P. lophurae* and into the wider environment either before or after inserting into a DNA virus vector. Notably, research on malaria was prioritized in the United States during World War II, and *P. lophurae* stocks were distributed to laboratories throughout the country (see Table S6) for experimental vaccine and drug research. During this period the poultry industry was scaling rapidly, and the first avian virus vaccines were being commercially developed (including live FWPV vaccines, based on attenuated virus strains grown in embryonated eggs [50],[51]). REV sequences have been reported in FWPV vaccines lyophilized in 1949 [52], suggesting that insertion had already occurred by this time. Unfortunately, however, this inference is subject to some incertitude, since it is based solely on PCR from a single archived sample, and no lyophilized material remains for study (Table 2).

The creation of Mareks disease vaccines became a priority in the United States during the 1950s, in response to devastating outbreaks of an apparently new, acute form of the disease [41]. However, effective vaccines were not produced until after the first avian cell culture systems were established in the 1960s. These *in vitro* systems were key to the eventual development of vaccines based on (i) attenuated GHV-2 strains and (ii) the closely related herpesvirus of turkeys (HVT). Both of these vaccines were later discovered to be contaminated with REV. In previous studies it has generally been assumed that REV insertions into the GHV-2 genome originated in the distant evolutionary past [3],[11],[12] (although it is recognized that at least some were generated recently during *in vitro* attenuation [53],[54]). By contrast, our data suggest that all REV insertions into GHV-2 have been generated recently.

In 1960s and 1970s REV provided an experimental model for retrovirologists [55], and was sometimes used to transform avian cells [56]. Thus it is likely that the emergence of avian cell culture systems was accompanied by the spread of REV as a contaminant. Interestingly, dissemination of REV genetic material appears to be ongoing; REV is apparently being maintained as an insertion in naturally circulating FWPV-REV, and field strains of GHV-2 containing novel REV LTR insertions have recently been reported [19],[20]. Furthermore, we show that the recently described exogenous REV isolate HA9901 [39] shares a common history with REV plasmid pREVA6, which was in turn derived from the original tissue sample from which prototypic REV strains were isolated [40] (Figure 5, Figure S2). Thus it appears that in China, REV-contaminated materials may have given rise to independently circulating infectious retrovirus. The processes driving REV dissemination warrant further exploration, as does the potential role of co-opted REV sequences in altering the *in vivo* properties of FWPV and/or GHV-2.

In conclusion, historical, phylogenetic, and paleovirological evidence supports a scenario wherein REVs originated as mammalian retroviruses that were iatrogenically introduced into avian hosts, and subsequently integrated into the FWPV and GHV-2 genomes, generating recombinant DNA viruses that now circulate in wild birds and poultry. These data provide the first evidence that horizontal gene transfer between virus families can expand the impact of iatrogenic transmission events, raising questions about the potential, unintended impacts of live, recombinant vector vaccines. Broader surveillance of viral genetic diversity should be prioritized, so that the unintended consequences of experimental procedures on viral ecology and evolution can be better assessed and limited.

## Materials and Methods

### Screening *in Silico*

PERL scripts and the BLAST+ program suite were used to perform *in silico* screening of sequence databanks for sequences homologous to REV. We screened complete and low coverage whole genome sequence data representing 10 avian and 42 mammalian species (Table S1) and all poxvirus and herpesvirus-derived sequence data available in GenBank as of July 1, 2012. The noncoding nucleotide sequences (LTR and leader) and translated open reading frames (ORFs) (Gag, Pol, Env) of REV (FJ439119.1) were used as “probes” for *in silico* screening. Sequences that matched probes with high statistical significance (i.e., expect (e) values 0.001) were extracted and compared to a library of reference retroviral genomes (see Table S2), again using BLAST. The results of this “reciprocal” BLAST were examined, and the phylogenetic relationships of ERV loci that disclosed higher similarity to REV than to any other retroviral reference were investigated using the neighbor joining (NJ) algorithm implemented in PAUP [57]. NJ trees revealed that among all the ERV loci identified by screening, ERVs in the European hedgehog (*Erinaceus europaeus*) and cape hyrax (*Procavia capensis*) genomes were most closely related to REV in the *gag-pol* and *env* genes, respectively (unpublished data). The median reciprocal BLAST bit score for these two subsets of ERVs was used to establish a threshold bit score for discriminating REV-related coding sequences from those of other ERVs.

### Tissue Samples, Virus, and Cell Culture

Frozen tissue samples from Malagasy carnivores (*Cryptoprocta ferox*, *Galidia elegans*, *Mungotictis decemlineata*) were obtained from the American Museum of Natural Historys cryogenic collection. Frozen spleen tissue samples were obtained from a deceased echidna (*Tachyglossus aculeatus*) at the Philadelphia Zoo. Chicken embryonic fibroblasts, SL-29 cells (ATCC#: CRL1590), were maintained in DMEM medium (Life technologies) supplemented with 5% fetal bovine serum, 5% tryptose phosphate broth, penicillin (100 U/ml), and streptomycin (100 mg/ml). An aliquot of 400 ul of DIAV (ATCC #: VR775) was inoculated onto 30% confluent SL-29 cells in six-well plates. Media was changed after 2 days, and cells were allowed to grow for a total of 5 days. After 5 days, cells were harvested and genomic DNA was extracted.

### PCR and Sequencing

Genomic DNA was extracted from tissue samples and SL-29 cells using the AllPrep dual DNA/RNA extraction kit (QIAGEN). Initial PCR amplification of endogenous retroviral fragments was performed using PCR primers (Integrated DNA Technologies) directed against two highly conserved motifs in retroviral protease (PR) and RT proteins. After initial sequencing of this genomic region, a combination of gene-specific primers and degenerate primers were used to amplify the remaining regions of the REV genomes found in *Galidia elegans*, *Mungotictis decemlineata*, and *Tachyglossus aculeatus*. LTR regions and genomic insertion sites were amplified and cloned by ligation-mediated PCR, using the GenomeWalker Universal kit (Clontech). For complete genome sequencing of DIAV, primers were based on equivalent targets in REV and SNV and were used to amplify multiple overlapping regions of the DIAV genome. A list of primer sequences, the genomes on which they were used, and their coordinates (based on alignment to the DIAV genome) can be found in Table S3. Basic PCR conditions were used for almost all reactions (denaturation at 95°C for 2 min, followed by 30 cycles of 94°C for 15 s, 55°C for 30 s, and 68°C for 1 min, final elongation for 10 min), although annealing temperatures and elongation times varied depending on the primers used (details available on request). For all reactions, gel-resolved amplicons were excised from 1% agarose gels and purified using the Qiaquick kit (QIAGEN) before TA cloning into pCR2.1 (Life Technologies, La Jolla, CA) and sequencing. All sequence analysis was performed by the GeneWiz commercial sequencing facility (GeneWiz, South Plainfield, NJ). Sequences obtained in this study have been submitted to Genbank under the following accession numbers: DIAV (KF313137); Echidna-ERV (KF313136), and *Galidia*-ERV (KF313135).

### Sequence Data and Phylogenetic Analyses

Retroviral “pan-genus” phylogenies were constructed from an alignment of the highly conserved RT and transmembrane (TM) peptides. Sequences derived from the retroviral reference library (Table S2) were included, as well as a selection of the best matching, uncharacterized ERVs from *in silico* and PCR screening. For both genes, ProtTest was used to select the best fitting amino acid substitution matrix from a range of 96 different combinations of models and rate heterogeneity parameters, based on the Akaike information criterion (AIC) [58]. The best fitting model for RT was rtREV [59], with gamma distributed rate heterogeneity (rtREV+Γ); for TM it was HIVw [60].

Phylogenetic investigation of within-REV variation was conducted using both peptide and nucleotide sequence data. We obtained all published REV sequence data from Genbank. Sequences shorter than 100 bp were excluded. The location and year of sampling, and host species associations, were extracted from the Genbank file or from an associated publication (Table S4). All sequences were profile aligned to a full genome reference (SNV; DQ003951.1). So that the phylogenetic relationships of all available sequences could be investigated, phylogenies were constructed for a range of alignment partitions: (i) complete genome, (ii) LTRs, (iii) *gag*, (iv) *pol*, and (v) *env*. Each partition was examined for evidence of recombination using GARD [61] and SplitsTree [62]. One full genome sequence (GQ375848.1) appeared to be recombinant and was subsequently removed from our dataset. We used ProtTest and ModelTest to select the best fitting amino acid and/or nucleotide substitution matrices for each alignment partition. The best fitting model for all nucleotide alignments was the general time reversible model [63] with a proportion of invariable sites and a gamma-shaped rate variation across sites (GTR+I+G). The best fitting models for amino acid alignments were; Gag, JTT+I; Pol, FLU+G; Env, JTT. The ML phylogeny was constructed using RAXML [64], with 1,000 nonparametric bootstrap replicates. A REV ancestral sequence was reconstructed using PAML [65].

### Literature Review

To systematically review REV-related literature, electronic searches of PubMed/Medline, JSTOR, Mendley, Scopus, Web of Science, and WorldCat were conducted in July 2012. Keywords used to search databases were “Reticuloendotheliosis Virus,” “Duck Infectious Anemia Virus,” “Spleen Necrosis Virus,” and “Chick Sync[i/y]tial Virus.” We restricted our search to papers with titles and abstracts available in English. The following data were searched for in texts: year of virus isolation, virus association, origin of isolation, animal status, secondary disease association, place of isolation, and the methods of isolation or detection. A completed PRISMA checklist and flow diagram for this systematic literature review can be found in Text S1.

## Supporting Information

Figure S1 **A nucleotide alignment of orthologous ERV insertion sites in the** ***Galidia elegans*** **(** ***Galidia*** **-ERV-1,2) and** ***Mungotictis decemlineata*** **(** ***Mungotictis*** **-ERV-1,2) genomes.** The alignment illustrated spans the 3′LTR and 3′ end of *env* of the orthologous REV-related ERV insertion in these two species and 238 bp of flanking genomic DNA (shown in grey). Flanking DNA is shown aligned to a homologous genomic sequence identified in the cat (*Felis catus*), dog (*Canis familiaris*), panda (*Ailuropoda melanoleuca*), and ferret (*Mustela furo*) genomes.
(PDF)

Figure S2 **An alignment of REV LTR sequences, showing the presence of unique shared indels (insertions and deletions) that support the monophyletic relationship of the three sequences highlighted in gray, which include (i) the HA9901 strain of REV, (ii) REV plasmid (pREVA6), and (iii) a REV LTR insertion present in the JM-Hi3 strain of GHV-2.** Shared indels are indicated by boxes.
(PDF)

Table S1 **Avian and mammalian whole genome sequences screened for REV-related ERVs.**
(DOCX)

Table S2 **Retroviral reference sequences used in the study.** Annotated reference sequences representing newly described ERVs have been made available online (http://saturn.adarc.org/paleo/).
(DOCX)

Table S3 **Primer Coordinates.** ^a^The coordinates of primers are shown, based on alignment to DIAV reference sequence (Accession Number KF313137). Abbreviations: F, forward; R, reverse; REV, reticuloendotheliosis virus; LTR, long terminal repeat.
(DOCX)

Table S4 Abbreviations: REV, reticuloendotheliosis virus; FWPV-REV, fowlpox virus with REV insertion; GHV-2-REV, gallid herpesvirus type 2 with REV insertion; LTR, long terminal repeat.
(DOCX)

Table S5 ^a^State or prefecture/two-letter ISO country code.
(DOC)

Table S6 **Asterisks indicate studies sponsored by the US Office of Scientific Research and Development (OSRD).**
(DOC)

Text S1 **PRISMA checklist and flow diagram.**
(PDF)

## Abbreviations

DIAV: duck infectious anemia virus
ERV: endogenous retrovirus
FWPV: fowlpox virus
GHV-2: gallid herpesvirus type 2
ICT: interclass transmission
LTR: long terminal repeat
Ma: million years ago
PCR: polymerase chain reaction
REV: reticuloendotheliosis virus
RT: reverse transcriptase
SNV: spleen necrosis virus
TM: transmembrane protein
