# Supplementary material for: The Extraordinary Evolutionary History of the Reticuloendotheliosis Viruses
Source: PLoS Biol. 2013 Aug 27;11(8):e1001642. doi: 10.1371/journal.pbio.1001642 (PMC3754887; doi:10.1371/journal.pbio.1001642)
Supplement: Figure S1 — A nucleotide alignment of orthologous ERV insertion sites in the Galidia elegans ( Galidia -ERV-1,2) and Mungotictis decemlineata ( Mungotictis -ERV-1,2) genomes. The alignment illustrated spans the 3′LTR and 3′ end of env of the orthologous REV-related ERV insertion in these two species and 238 bp of flanking genomic DNA (shown in grey). Flanking DNA is shown aligned to a homologous genomic sequence identified in the cat (Felis catus), dog (Canis familiaris), panda (Ailuropoda melanoleuca), and ferret (Mustela furo) genomes. (PDF) [file pbio.1001642.s001.pdf]

Fig. S1

```
                    env
Ge-ERV-1    GACTCCCTCGCTGAGGTGGTCCTCCAAAATCGGAGGGGACTTGACCTGCTCACGGCAGAG 60
Ge-ERV-2    ...............................................A............
Md-ERV-1    ...............................................G............
Md-ERV-2    ...............................................A............

Ge-ERV-1    GAAAAAAAATCTGCCTGGCCTTACAGGAACGCTGCTACTTCTATGCCATATGAATCAAAG 120
Ge-ERV-2    ............................................................
Md-ERV-1    ........-...................................................
Md-ERV-2    ............................................................

            env end                  3' LTR start
Ge-ERV-1    ATTTGATTCATAAACTCCAAAGCAGTGGGGAATGTAACAGGGGGGATATGGCAAGGGGTG 180
Ge-ERV-2    ............................................................
Md-ERV-1    ............................................................
Md-ERV-2    ............................................................

Ge-ERV-1    AAGGGATGTTGCAAGAGGCAAGAGGCATGTGACTACAAATGTGGCAGGGGATGTTGCAAG 360
Ge-ERV-2    ............................................................
Md-ERV-1    ............................................................
Md-ERV-2    ............................................................

Ge-ERV-1    AGGCAAGAGGCATGTGACTAAGAATGTGGCAGGGGATGTTGCAAGAGGCATGTGACTAAG 420
Ge-ERV-2    ............................................................
Md-ERV-1    ............................................................
Md-ERV-2    ............................................................

Ge-ERV-1    GATGTGACAAGGGATGTTGCAAGAGGCAAGGGGCATGTGACTAAGAATG----------- 480
Ge-ERV-2    .................................................-----------
Md-ERV-1    .................................................-----------
Md-ERV-2    .................................................TGGCAGGGGAT

                         3' LTR end
Ge-ERV-1    ---------------------------TGGCAAGGAATGTTGCAAGAGGCATGTGACTAG 540
Ge-ERV-2    ---------------------------.................................
Md-ERV-1    ---------------------------.................................
Md-ERV-2    GTTGCAAAGGGCATATGACTAAGGATA.................................

Ge-ERV-1    GAATGTGACAAGGGATGTTGCAAAAGGCATGTGACTAAGGATATGGCAAAGGATGTTACA 600
Ge-ERV-2    ............................................................
Md-ERV-1    ............................................................
Md-ERV-2    ............................................................

Ge-ERV-1    AGGGGCATATGACTAAAGATATAGCAAGGGGTCTTGCAACAAAAGTCACATAGCCACCCC 660
Ge-ERV-2    ............................................................
Md-ERV-1    ............................................................
Md-ERV-2    ............................................................
```

```
Ge-ERV-1        TTAGAAGAGAATAAAAGAGGAGGATGGAGAAGGCTAAGTGCTGCTCATACCTGCTACCCG 720
Ge-ERV-2        ............................................................
Md-ERV-1        ............................................................
Md-ERV-2        ............................................................

Ge-ERV-1        CTTTTGCTTAGCAGGGCGTCAGCCCAGATTCGAATCTGTAATAAATTAAAAAAAATCCCA 780
Ge-ERV-2        ............................................................
Md-ERV-1        ............................................................
Md-ERV-2        ............................................................

                                                     insertion site
                                                     --->  <---
Ge-ERV-1        AATGCTCTGGGGTCGTCTGTCCTTGCAGGGTCTTCACTTCATTACAGTATTGTTATTTGG 840
Ge-ERV-2        ............................................................
Md-ERV-1        ............................................................
Md-ERV-2        ............................................................
Cat                                                           ..............
Dog                                                           ..............
Panda                                                         .......G......
Ferret                                                        .......G......

Ge-ERV-1        GCACTTTTAAAAGTTTTAAAAAAACATATTTTCTCC------------TTAGTTACACAA 900
Ge-ERV-2        ....................................------------............
Md-ERV-1        ....................................------------............
Md-ERV-2        ....................................------------............
Cat             ........GAAAGT.....GGG.GTG.GTTTTCTCCTGAATTTTCTCC.AG...GA.G..
Dog             ........GAAAG-.....GGG.G.A.GTTTTCTTC----TTTTCTCA.AG......G..
Panda           ........GAAAG-.....GGG.G.A.GTTTTCTCC----TTTTCTCA.AG......G..
Ferret          ........G....-.....GCG.G............----TTTTGTCA.AG..CTC....

Ge-ERV-1        TTGTTGCCTATGGTTCTATAATTCTGATCAACTTCTTAAAAGCCTTTCTGTGCTTCAGAG 960
Ge-ERV-2        ............................................................
Md-ERV-1        ............................................................
Md-ERV-2        ............................................................
Cat             .....AG..GCA......GG...T..........T........TA..........TG..T
Dog             .....A...GCA.......G...T..........T.........A..........TG..T
Panda           .....A...GCA.......G...T..........T.........A..........TG..T
Ferret          ..T..A...GC..-....GG...T..........T..---....A..........TG..T

Ge-ERV-1        TTATATAAAAGAAGATAC----ATTCACAATATTATGCACACTGACTACAAGCTAGTTAG 1020
Ge-ERV-2        ..................----......................................
Md-ERV-1        ..................----......................................
Md-ERV-2        ..................----......................................
Cat             ..G.G.G....G..G.G.----GA.TT..GC...G.T..TG........G.T.....G..
Dog             .GG.G.G....G.TG..TGATCGA...T.GC.G.C.T.GTC...G..............T
Panda           .CG.G.G....G.TG.GT----GA....GGC...G.T..TG...G..........C...T
Ferret          CCG.G.G....G.TG.GT----GA....GGC...G.T..TG...G...T.....G....T

                --->
Ge-ERV-1        CAC 1024
Ge-ERV-2        CAC
Md-ERV-1        CAC
Md-ERV-2        CAC
Cat             CTT
Dog             CTT
Panda           CTT
Ferret          TTT
```
