# Supplementary material for: The Extraordinary Evolutionary History of the Reticuloendotheliosis Viruses
Source: PLoS Biol. 2013 Aug 27;11(8):e1001642. doi: 10.1371/journal.pbio.1001642 (PMC3754887; doi:10.1371/journal.pbio.1001642)
Supplement: Figure S2 — An alignment of REV LTR sequences, showing the presence of unique shared indels (insertions and deletions) that support the monophyletic relationship of the three sequences highlighted in gray, which include (i) the HA9901 strain of REV, (ii) REV plasmid (pREVA6), and (iii) a REV LTR insertion present in the JM-Hi3 strain of GHV-2. Shared indels are indicated by boxes. (PDF) [file pbio.1001642.s002.pdf]

Fig. S2

```
SNV            TGTGGGAGGG AGCTCTGGGG GGAATAGTGC TGGCTCGCTA ACTGCTATAT TAGCTTCTGT ACCCATGCTT GCTTGCCTGG CCACTAACCG CCATATTAGC TTCTGTACAC ATGCTTGCTT 120
DIAV           .......... .....C.... .......C.. .......... .......... .......... ..AT...... ........-- ---------- ---------- ---------- ----------
REV-HA9901     .......... .....C.... .......C.. .......... .....CG... .......... .AT....... ........-- ---------- ---------- ---------- ----------
pREVA6         .......... .....C.... .......C.. .......... .....C.... .......... .AT....... ........-- ---------- ---------- ---------- ----------
GHV2 JM-Hi3    .......... .....C.... .......C.. .......... .....C.... .......... .AT....... ........-- ---------- ---------- ---------- ----------
REV-3410/06    .......... .....C.... .......C.. .......... .....C.... .......... .AT....... ........-- ---------- ---------- ---------- ----------
REV-33337/05   .......... .....C.... .......C.. .......... .....C.... .......... .AT....... ........-- ---------- ---------- ---------- ----------
REV-ZD0708     .......... .....C.... .......C.. .......... .....C.... .......... .AT....... ........-- ---------- ---------- ---------- ----------
REV-APC        .......... .....C.... .......C.. .......... .....C.... .......... .AT....... ........-- ---------- ---------- ---------- ----------
REV-HLJR0901   .......... .....C.... .......C.. .......... .....C.... .......... .AT....... ........-- ---------- ---------- ---------- ----------
FWPV-IL field  .....-.... .....C.... .......C.. .......... .....C.... .......... .AT....... ........-- ---------- ---------- ---------- ----------

SNV            GCCTTAGCCG CCATTGTACT TGATATGCCA TTTCTCGGAA TCGGCATCAA GTCTCGCTTC TCGGAATCGG CATCAAGTTT CGCTTCTCGA AATCGGCGTC ATTTCTCGGC ATCGAGAGCA 240
DIAV           ----C..... ....AA...C .A........ .......... .......... .......... ........A. .......... .....T.... ....A..... .......... ..........
REV-HA9901     ----...... .......... ......---. ....G.T..- ---------- -.A..AT... ........A. .....--... .T.----G.. .--------- -----..... ...A......
pREVA6         ----...... .......... ......---. ....G.T..- ---------- -.A..AT... .......... .....--... .T.----G.. .--------- -----..... ...A......
GHV2 JM-Hi3    ----...... .......... ......---. ....G.T..- ---------- -.A..AT... .......... .....--... .T.----G.. .--------- -----..... ...A......
REV-3410/06    ----...... .......... ......---. ....G.T..- ---------- -.A..AT... .......... .....----- ---------- ---------- ---------- ----......
REV-33337/05   ----...... .......... ......---. ....G.T..- ---------- -.A..AT... .......... .....----- ---------- ---------- ---------- ----......
REV-ZD0708     ----...... .......... ......---. ....G.T..- ---------- -.A..AT... .......... .....----- ---------- ---------- ---------- ----......
REV-APC        ----...... .......... ......---. ....G.T..- ---------- -.A..AT... .......... .....----- ---------- ---------- ---------- ----......
REV-HLJR0901   ----...... .......... ......---. ....G.T..- ---------- -.A..AT... .......... .....----- ---------- ---------- ---------- ----......
FWPV-IL field  ----...... .......... ......---. ....G.T..- ---------- -.A..AT... .......... .....----- ---------- ---------- ---------- ----......

SNV            AGCTCATAAA CCATAAAAGG AAATGTGTAT TGAAGGCAAG CATCAGACCA CTTGCGCCAT CCAATCACGA ACGAACACGA GATCGGACTA TCATACTGAG CCAATGGTTG TAAAGGGCAG 360
DIAV           ......C... .......... G.......TG .--------- -......... .......... .......... .......... .......... .......... ......T... ..........
REV-HA9901     G......G.. .......... ......T.G. .......... ......G... .......... .......... G.A....... .....A.... .......... .......... ..........
pREVA6         G......G.. .......... ......TCG. ..G...G... .......... .......... .......... .GA....... .....A.... .......... .......... ..........
GHV2 JM-Hi3    G......G.. .......... ......T.G. ..G...N... .......... .......... .......... G.A....... .....A.... .......... .......... ..........
REV-3410/06    G......... .......... ......T.G. .......... .......... .....A.... .......... ..A....... .....A.... .......... .......... ..........
REV-33337/05   G......... .......... ......T.G. .......... .......... .....A.... .......... ..A....... .....A.... .......... .......... ..........
REV-ZD0708     G......... .......... ......T.G. .......... .......... .....A.... .......... ..A....... .....A.... .......... .......... ..........
REV-APC        G......... .......... ......T.G. .......... .......... .....A.... .......... ..A....... .....A.... .......... .......... ..........
REV-HLJR0901   G......... .......... ......T.G. .......... .......... .....A.... .......... ..A....... .....A.... .......... .......... ..........
FWPV-IL field  G......... .......... ......T.G. .......... .......... .....A.... .......... ..A....... .....A.... .......... .......... ..........

                                                                                                              U3 | R
SNV            ATGCTACTCT CCAATAAGGG AAAATGTCAT GTAACACCCT GTAAGCTGTA AGCGGCTATA TAAGCCGGGT ACATCTCTTG CTCGGGGTCG CCGTCCTACA CATTGTTGTT GTGACGTGCG 480
DIAV           ..A..G.... .....G.... .........C .......... .......... .......... .......A.A ..C....... .......... .......... .......... ..........
REV-HA9901     ......TC.. .....G.... .......... .C....T... ..--C..... .......... ......A... G..C...... .......... .......... .........- --........
pREVA6         ......TC.. .....G.... .......... .C....T... ..--C..... .......... ......A... G......... .......... .......... .........- --....C...
GHV2 JM-Hi3    ......TC.. .....G.... .......... .C....T... ..--C..... .......... ......A... G......... .......... .......... .........- --........
REV-3410/06    ......TC.. .....G.... .......... .C....T.-- -----..... .......... ......A... G......... .......... .......... .........- --........
REV-33337/05   ......TC.. .....G.... .......... .C....T.-- -----..... .......... ......A... G......... .......... .......... .........- --........
REV-ZD0708     ......TC.. .....G.... .......... .C....T.-- -----..... .......... ......A... G......... .......... .......... .........- --........
REV-APC        ......TC.. .....G.... .......... .C....T.-- -----..... .......... ......A... G......... .......... .......... .........- --........
REV-HLJR0901   ......TC.. .....G.... .......... .C....T.-- -----..... .......... ......A... G......... .......... .......... .........- --........
FWPV-IL field  ......TC.. .....G.... .......... .C....T.-- -----..... .......... ......A... G......... .......... .......... .........- --........

                                                    R | U5
SNV            GCCCAGATTC GAATCTGTAA TAAAACTTTT TTTTTCTGAA TCCTCAGATT GGCAGTGAGA GGAGATTTTG TTCGTGGTGT TGGCTCGCCT ACTGGGTGGG CGCAGGGATC CGGACTGAAT 600
DIAV           .......... .......... .......... ..C....... .......... .......... .......... .......... .......... .......... T......... ..........
REV-HA9901     .......... .......... .....G.... C.C....AT. .......... .......... .......... .......... A....G.... .......... -.TT...... ..........
pREVA6         .......... .......... .....G.... ..C....AT. .......... .......... .......... .......... A....G.... .......... -.T....G.. ..........
GHV2 JM-Hi3    .......... .......... .....T.... ..C....AT. .......... .....GT... .......... .......... A....G.... .......... -.T....... ..........
REV-3410/06    .......... .......... .....GC... ..C....AT. .......... .......... .......... .......... .....G.... .......... -.T....... ..........
REV-33337/05   .......... .......... .....GC... ..C....AT. .......... .......... .......... .......... .....G.... .......... -.T....... ..........
REV-ZD0708     .......... .......... .....GC... ..C....AT. .......... .......... .......... .......... .....G.... .......... -.T....... ..........
REV-APC        .......... .......... .....GC... ..C....AT. .......... .......... .......... .......... .....G.... .......... -.T....... ..........
REV-HLJR0901   .......... .......... .....GC... ..C....AT. .......... .......... .......... .......... .....G.... .......... -.T....... ..........
FWPV-IL field  .......... .......... .....GC... ..C....AT. .......... .......... .......... .......... .....G.... .......... -.T....... ..........

SNV            CCGTAGTACT TCGG 614
DIAV           .......... ....
REV-HA9901     ......A.T. ..T.
pREVA6         ........T. ...A
GHV2 JM-Hi3    ........T. ..T.
REV-3410/06    ........T. ....
REV-33337/05   ........T. ....
REV-ZD0708     ........T. ....
REV-APC        ........T. ....
REV-HLJR0901   ........T. ....
FWPV-IL field  ........T. ....
```
