# Supplementary material for: The Extraordinary Evolutionary History of the Reticuloendotheliosis Viruses
Source: PLoS Biol. 2013 Aug 27;11(8):e1001642. doi: 10.1371/journal.pbio.1001642 (PMC3754887; doi:10.1371/journal.pbio.1001642)
Supplement: Table S1 — Avian and mammalian whole genome sequences screened for REV-related ERVs. (DOCX) [file pbio.1001642.s003.docx]

**Table S1.** Avian and mammalian genome sequences screened

| **Species name** | **Common name** | **Order** | **Version** |
| --- | --- | --- | --- |
| **Class Aves** | | | |
| *Gallus gallus* | Chicken | Galliformes | NCBI 2.1 November 2006 |
| *Meleagris gallopavo* | Turkey | Galliformes | NCBI 1.1 May 2011 |
| *Taeniopygia guttata* | Zebrafinch | Passeriformes | NCBI 1.1 March 2009 |
| *Columba livia* | Rock dove | Columbiformes | BGI 1.1 April 2013 |
| *Falco cherrug* | Saker falcon | Falconiformes | BGI 1.1 April 2013 |
| *Falco peregrinus* | Peregrine falcon | Falconiformes | BGI 1.1 April 2013 |
| *Geospiza fortis* | Medium ground finch | Passeriformes | BGI 1.1 April 2013 |
| *Melopsittacus undulatus* | Budgerigar | Psittaciformes | WUSTL, 6.3 April 2013 |
| *Anas platyrhynchos* | Mallard | Anseriformes | BGI 1.1 April 2013 |
| *Pseudopodoces humilis* | Ground-tit | Passeriformes | BGI 1.1 April 2013 |
|  |  |  |  |
| **Class Mammalia** | | | |
| *Microcebus murinus* | Grey mouse lemur | Primates | EMBL 1.54 June 2009 |
| *Tarsius syrichta* | Tarsier | Primates | EMBL 1.54 June 2009 |
| *Otolemur garnettii* | Bush baby | Primates | EMBL 1.54 June 2009 |
| *Callithrix jacchus* | Common marmoset | Primates | WUSTL 1.0 2009 |
| *Nosomascus leucogenys* | Northern white-cheeked gibbon | Primates | EBI Nleu 1.0 June 2011 |
| *Macaca mulatta* | Rhesus macaque | Primates | NCBI 1.1 June 2006 |
| *Papio hamadryas* | Hamadryas baboon | Primates | Baylor 1.0 June 2009 |
| *Pongo pygmaeus* | Orang-utan | Primates | EMBL 2.54 June 2009 |
| *Gorilla gorilla* | Gorilla | Primates | EMBL 1.54 June 2009 |
| *Pan troglodytes* | Chimpanzee | Primates | NCBI 2.1 October 2006 |
| *Homo sapiens* | Human | Primates | NCBI 36.3 March 2008 |
| *Vicugna pacos* | Alpaca | Artiodactyla | EMBL 1.54 June 2009 |
| *Bos taurus* | Cow | Artiodactyla | NCBI 4.1 August 2008 |
| *Sus scrofa* | Pig | Artiodactyla | NCBI 1.1 July 2008 |
| *Ovis aries* | Sheep | Artiodactyla | EBI Version 1 June 2011 |
| *Equus caballus* | Horse | Perissodactyla | NCBI 2.1 July 2008 |
| *Ailuropoda melanoleuca* | Panda | Carnivora | BGI Feb 2010 |
| *Canis familiaris* | Domestic dog | Carnivora | NCBI 2.1 September 2005 |
| *Felis catus* | Domestic cat | Carnivora | EMBL 1.54 June 2009 |
| *Mustela furo* | Ferret | Carnivora | Broad 1.1 June 2011 |
| *Sorex araneus* | Common shrew | Soricomorpha | EMBL 1.54 June 2009 |
| *Pteropus vampyrus* | Flying fox | Chiroptera | EMBL 1.54 June 2009 |
| *Myotis lucifugus* | Little brown bat | Chiroptera | EMBL 1.54 June 2009 |
| *Cavia porcellus* | Guinea pig | Rodents | EMBL 3.54 June 2009 |
| *Spermophilus tridecemlineatus* | Thirteen lined ground squirrel | Rodents | EMBL 1.54 June 2009 |
| *Mus musculus* | Mouse | Rodents | NCBI 37.1 July 2007 |
| *Rattus norvegicus* | Brown rat | Rodents | NCBI 4.1 June 2006 |
| *Dipodomys ordii* | Kangaroo rat | Rodents | EMBL 1.54 June 2009 |
| *Ochotona princeps* | Pika | Lagomorpha | EMBL 1.54 June 2009 |
| *Oryctolagus cuniculus* | European rabbit | Lagomorpha | EMBL 1.54 June 2009 |
| *Loxodonta africana* | African elephant | Proboscidea | EMBL 2.54 June 2009 |
| *Tursiops truncatus* | Bottlenose dolphin | Cetacea | Baylor 1.0 June 2009 |
| *Procavia capensis* | Cape hyrax | Hyracoidea | EMBL 1.54 June 2009 |
| *Erinaceus europaeus* | European hedgehog | Erinaceomorpha | EMBL 1.54 June 2009 |
| *Echinops telfari* | Lesser hedgehog tenrec | Afrosoricida | EMBL 1.54 June 2009 |
| *Tupaia belangeri* | Tree shrew | Scandentia | EMBL 1.54 June 2009 |
| *Choloepus hoffmanni* | Hoffmann’s three toed sloth | Pilosa | EMBL 1.54 June 2009 |
| *Dasypus novemcinctus* | Nine-banded armadillo | Cingulata | EMBL 2.54 June 2009 |
| *Monodelphis domestica* | Oppossum | Didelphimorphia | NCBI 2.1 March 2007 |
| *Macropus eugenii* | Tammar wallaby | Diprotodontia | Baylor 1.1 June 2009 |
| *Ornithorhynchus anatinus* | Duck-billed platypus | Monotremata | NCBI 1.1 July 2007 |
| *Sarcophilus harrisii* | Tasmanian devil | Dasyuromorphia | EBI Devil 7.0 Oct 2011 |
