# Supplementary material for: The Extraordinary Evolutionary History of the Reticuloendotheliosis Viruses
Source: PLoS Biol. 2013 Aug 27;11(8):e1001642. doi: 10.1371/journal.pbio.1001642 (PMC3754887; doi:10.1371/journal.pbio.1001642)
Supplement: Table S2 — Retroviral reference sequences used in the study. Annotated reference sequences representing newly described ERVs have been made available online (http://saturn.adarc.org/paleo/). (DOCX) [file pbio.1001642.s004.docx]

**Table S2.** Retroviral reference sequence library

| **Virus name** | **Abbrev.** | **Accession** |
| --- | --- | --- |
|  |  |  |
| **Genus Alpharetrovirus** |  |  |
| Avian leukemia virus-A | ALV-A | M37980.1 |
| Avian leukemia virus-J | ALV-J | Z46390.1 |
| **Genus Betaretrovirus** |  |  |
| Bovine endogenous retrovirus | BERV-beta3 | EF030818.1 |
| Jaagsiekte sheep retrovirus | JSRV | M80216.1 |
| Mouse mammary tumor virus | MMTV | M15122.1 |
| Mason Pfizer monkey virus | MPMV | M12349.1 |
| Squirrel monkey retrovirus | SMRV-H | M23385.1 |
| Simian retrovirus 1 | SRV-1 | M11841.1 |
| Simian retrovirus 2 | SRV-2 | M16605.1 |
| Simian retrovirus 4 | SRV-4 | M16605.1 |
| *Trichosurus vulpecula* endogenous retrovirus | TvERV | AF284693.1 |
| **Genus Deltaretrovirus** |  |  |
| Bovine leukemia virus | BLV | K02120.1 |
| Human T-cell leukemia virus 1 | HTLV-1 | J02029.1 |
| Human T-cell leukemia virus 2 | HTLV-2 | M10060.1 |
| Human T-cell leukemia virus 3 | HTLV-3 | DQ093792.1 |
| Human T-cell leukemia virus 4 | HTLV-4 | EF488483.1 |
| Simian T-cell leukemia virus 1 | STLV-1 | AY590142.1 |
| **Genus Epsilonretrovirus** |  |  |
| Walleye dermal sarcoma virus | WDSV | AF033822.1 |
| **Genus Gammaretrovirus** |  |  |
| Baboon endogenous retrovirus | BAEV | AHZZ01047987.1 |
| Feline leukemia virus | FeLV | M18247.1 |
| Human endogenous retrovirus T | HERV-T | AC247036.3 |
| Human endogenous retrovirus E | HERV-E | AC008265.1 |
| Gibbon ape leukemia virus | GaLV | M26927.1 |
| Koala retrovirus | KoRV | AF151794.2 |
| Killer whale endogenous retrovirus | KwERV | GQ222416.1 |
| Murine leukemia virus (Moloney) | MLV (Moloney) | J02255.1 |
| Murine leukemia virus (Graffi) | MLV (Graffi) | AB187566.2 |
| Murine leukemia virus (AKV) | MLV (AKV) | J01998.1 |
| Murine leukemia virus (Rauscher) | MLV (Rauscher) | U94692.1 |
| Murine leukemia virus (Friend) | MLV (Friend) | Z11128.1 |
| Porcine endogenous retrovirus A | PERV-A | EU789636.1 |
| Porcine endogenous retrovirus C | PERV-C | HM159246.1 |
| Porcine endogenous retrovirus E | PERV-E | NC_003059.1 |
| RD114 endogenous retrovirus | RD114 | NC_009889.1 |
| Reticuloendotheliosis virus | REV | FJ439119.1 |
| *Rhinolophus ferrumequinum* retrovirus | RfRV | JQ303225.1 |
| Wooly monkey sarcoma virus | WMSV | NC_009424.4 |
| Xenotropic murine leukemia virus-related retrovirus | XMRV | HQ154630 |
| **Genus Lentivirus** |  |  |
| Bovine immunodeficiency virus | BIV | M32690.1 |
| Equine infectious anemia virus | EIAV | M16575.1 |
| Feline immunodeficiency virus | FIV | M25381.1 |
| Feline immunodeficiency virus-puma | FIVpcc | U03982.1 |
| Human immunodeficiency virus 1 | HIV-1 | U26942 |
| Human immunodeficiency virus 2 | HIV-2 | M30502.1 |
| Jembrana disease virus | JDV | U21603.1 |
| Simian immunodeficiency virus –African green monkey | SIVagm | M30931.1 |
| Simian immunodeficiency virus –Colobus | SIVcol | AF301156.1 |
| Simian immunodeficiency virus –Chimpanzee | SIVcpz | X52154.1 |
| Small ruminant lentivirus A | SRLV-A | M60609.1 |
| Small ruminant lentivirus B | SRLV-B | M33677.1 |
| **Genus Spumavirus** |  |  |
| Bovine foamy virus | BFV | U94514.1 |
| Equine foamy virus | EFV | AF201902.1 |
| Feline foamy virus | FFV | Y08851.1 |
| Simian foamy virus – Chimpanzee | SFVcpz | NC001364.1 |
| **Avian ERV-H** |  |  |
| Taeniopygia-ERV-H | Taeniopygia-ERV-H | ABQF01078579.1 |
| Zonotrichia-ERV-H | Zonotrichia-ERV-H | ARWJ01030705.1 |
| Falco-ERV-H | Falco-ERV-H | AKMT01021606.1 |
| Melopsittacus-ERV-H | Melopsittacus-ERV-H | AGAI01060520.1 |
| Columba-ERV-H | Columba-ERV-H | AKCR01081075.1 |
| Pseudopoces-ERV-H | Pseudopoces-ERV-H | ANZD01021539.1 |
| Geospiza-ERV-H | Geospiza-ERV-H | AKZB01052587.1 |
| **Avian ERV-R** |  |  |
| Zonotrichia-ERV-R | Zonotrichia-ERV-R | ARWJ01027361.1 |
| RV-rook | RV-rook | AJ236130.1 |
| Melopsittacus-ERV-R | Melopsittacus-ERV-R | AGAI01044309.1\| |
| Taeniopygia-ERV-R | Taeniopygia-ERV-R | ABQF01096890.1 |
| Anas-ERV-R | Anas-ERV-R | ADON01151600.1 |
| Gallus-ERV-R | Gallus-ERV-R | AADN03003986.1 |
| **Other class I ERVs** |  |  |
| ChiRV1 | ChiRV1 | DQ280312.2 |
| RV-komodo dragon | RV-komodo dragon | Y07807.1 |
| RV-lemon shark | RV-lemon shark | Y07810.1 |
| RV rock wallaby | RV rock wallaby | Y07809.1 |
| RV-sparrow | RV-sparrow | Y07808.1 |
| Human endogenous retrovirus XA | HERV-XA | AC114321.2 |
| Human endogenous retrovirus Fb | HERV-Fb | AC002416 |
| Human endogenous retrovirus H | HERV-H | K01891 |
| Endogenous retrovirus Fc | ERV-Fc | AJ507118.1 |
| Endogenous retrovirus 9 | ERV-9 | AC107219.5 |
| Human endogenous retrovirus W | HERV-W | AF135487 |
| Human endogenous retrovirus I | HERV-I | M92067 |
| Gallus-ERV | Gallus-ERV | AADN03020210.1 |
| Meleagris-ERV | Meleagris-ERV | ADDD01127175.1 |
|  |  |  |
