# Supplementary material for: The Extraordinary Evolutionary History of the Reticuloendotheliosis Viruses
Source: PLoS Biol. 2013 Aug 27;11(8):e1001642. doi: 10.1371/journal.pbio.1001642 (PMC3754887; doi:10.1371/journal.pbio.1001642)
Supplement: Table S3 — Primer Coordinates. aThe coordinates of primers are shown, based on alignment to DIAV reference sequence (Accession Number KF313137). Abbreviations: F, forward; R, reverse; REV, reticuloendotheliosis virus; LTR, long terminal repeat. (DOCX) [file pbio.1001642.s005.docx]

**Table S3: Primer sequences used for PCR screening**

| **Primer name** | **Sequence (5'-3')** | **Direction** | **Target virus(es)** | **Target region** |
| --- | --- | --- | --- | --- |
| **AN_100002** | GTK TTI KTI GAY ACI GGI KC | F | Retroviruses | *pol* (2545-2564) |
| **AN_100003** | ATI AGI AKR TCR TCC ATR TA | R | Retroviruses | *pol* (3496-3515) |
| **AN_100004** | AGI AKR TCR TCC ATR TA | R | Retroviruses | *pol* (3496-3512) |
| **AN_100043** | CAC CAG CCY ACC TGG GAY GAY TG | F | Gammaretroviruses | *gag* (1731-1753) |
| **AN_100044** | GGT AGC TTT TGG AAA TCC CGG TGG C | R | Mongoose REV | *pol* (2628-2652) |
| **AN_100045** | GCC ACG ACC TCC AGA GTT ACC G | F | Mongoose REV | *pol* (3442-3463) |
| **AN_100046** | AAA GGC CGG TCC ATT GTC GGA | R | Gammaretroviruses | *pol* (5391-5411) |
| **AN_100086** | GTG GTG AAA AGG ATC CGG AGA AGT TGC T | R | Mongoose REV | *gag* (1756-1783) |
| **AN_100087** | GAG GCC TTC CCT ACA AAG AGA GAG ACA | F | Mongoose REV | *pol* (5295-5321) |
| **AN_100123** | RGG KTY AKR CAH AGC CAG CA | R | Gammaretroviruses | *env* (6755-6774) |
| **AN_100124** | CCN GAS TKR TTR GCR TAM AAR CAR CA | R | Gammaretroviruses | *env* (7448-7473) |
| **AN_100132** | AAC GGG CTT CTG CAC TAC TGA | R | Echidna REV | *pol* (2571-2591) |
| **AN_100133** | TCA GTA GTG CAG AAG CCC GTT | F | Echidna REV | *pol* (2571-2591) |
| **AN_100134** | AGC GTT ACT GTT GGG TGG TCA | R | Echidna REV | *pol* (3467-3487) |
| **AN_100135** | TGA CCA CCC AAC AGT AAC GCT | F | Echidna REV | *pol* (3467-3487) |
| **AN_100136** | CTC RGG TAA CAG GGT RGC CG | R | All REVs | *pol* (4273-4294) |
| **AN_100146** | GAC TCC CTC GCT GAG GTG GTC CTC CAA AAT CGG AGG GGA CTT GAC CTR CTC ACG GCA GAG | F | Mongoose REV | *env* (7355-7414) |
| **AN_100150** | CCG CTT TTG CTT AGC AGG GCG TCA GCC CAG ATT CGA ATC TGT | F | Mongoose REV | LTR (401-442) |
| **AN_100151** | ACA GAT TCG AAT CTG GGC TGA CGC CCT GCT AAG CAA AAG CGG | R | Mongoose REV | LTR (401-442) |
| **AN_100152** | GTC GTA AAC AAG ACG CGG AGG AG | R | Echidna REV | *gag* (1761-1783) |
| **AN_100153** | CCG CGA TTT GGA CTG CCA TTT | F | Echidna REV | *env* (5361-5381) |
| **AN_100154** | CCT CCG TCG TAA ACA AGA CGC GGA GGA GCT GCT GGC | R | Echidna REV | *gag* (1753-1788) |
| **AN_100155** | GCT GAC GGC TGA ACA GGG CGG GAT CTG CCT A | F | Echidna REV | *env* (7402-7432) |
| **AN_100156** | GTC GTA AAC AAG ACG CGG A | R | Echidna REV | *gag* (1765-1783) |
| **AN_100157** | GCC TAG CCC TAA AGG AAC ATT G | F | Echidna REV | *env* (7428-7449) |
| **AN_100158** | CRG CCC AGA TTC GAA TCT GTA ATA AA | F | All REVs | LTR (423-448) |
| **AN_100159** | TTT ATT ACA GAT TCG AAT CTG GGC YG | R | All REVs | LTR (423-448) |
| **AN_100178** | TGT GGG AGG GAG CTC YGG | F | DIAV | LTR (1-18) |
| **AN_100179** | CGG AGG AGT TGC TGG CAA TCA | R | DIAV | *gag* (1748-1768) |
| **AN_100180** | TGA TTG CCA GCA ACT CCT CCG | F | DIAV | *gag* (1748-1768) |
| **AN_100181** | TCC CGT TTC CAC TGC TCT AGC A | R | DIAV | *pol* (2894-2915) |
| **AN_100182** | TGC TAG AGC AGT GGA AAC GGG A | F | DIAV | *pol* (2894-2915) |
| **AN_100183** | CTG CTC TGG GAA GCA AGA GGC | R | DIAV | *pol* (4970-4990) |
| **AN_100184** | GCC TCT TGC TTC CCA GAG CAG | F | DIAV | *pol* (4970-4990) |
| **AN_100185** | GAG GGG TAG CTG TGC CTG ATG | R | DIAV | *env* (6611-6630) |
| **AN_100186** | CAT CAG GCA CAG CTA CCC CTC | F | DIAV | *env* (6611-6630) |
| **AN_100187** | TGT TGT ACC GAA VTA CKA CGG ATT CAG | R | *Galidinae* | LTR (8421-8447) |
| **AN_100189** | CGT TGT AAA ACG ACG GCC AGT GA | R | *Galidinae* | REV 3’LTR flank |
