# Supplementary material for: The Extraordinary Evolutionary History of the Reticuloendotheliosis Viruses
Source: PLoS Biol. 2013 Aug 27;11(8):e1001642. doi: 10.1371/journal.pbio.1001642 (PMC3754887; doi:10.1371/journal.pbio.1001642)
Supplement: Table S4 — Abbreviations: REV, reticuloendotheliosis virus; FWPV-REV, fowlpox virus with REV insertion; GHV-2-REV, gallid herpesvirus type 2 with REV insertion; LTR, long terminal repeat. (DOCX) [file pbio.1001642.s006.docx]

**Table S4: Previously published REV sequences**

| **Accession #** | **Virus source** | **Host species** | **Virus region** | **Isolation details** | |
| --- | --- | --- | --- | --- | --- |
|  |  |  |  | **Location** | **Year** |
| [AF006065.1](http://www.ncbi.nlm.nih.gov/nuccore/AF006065.1) | FWPV-REV | FWPV vaccine | *pol* | Australia | 1997 |
| [AF246698.2](http://www.ncbi.nlm.nih.gov/nuccore/AF246698.2) | FWPV-REV | FWPV in chickens | Full genome | IL, USA | 2000 |
| [AY255632.1](http://www.ncbi.nlm.nih.gov/nuccore/AY255632.1) | FWPV-REV | Vaccine strain TCP-Blen | LTR | KS/USA | 2003 |
| [AY842951.1](http://www.ncbi.nlm.nih.gov/nuccore/AY842951.1) | REV-HA9901 | Chicken | Full genome | Shandong, China | 2006 |
| [DQ003591.1](http://www.ncbi.nlm.nih.gov/nuccore/DQ003591.1) | Spleen necrosis virus (SNV) | Unknown | Full genome | Shandong, China (ex USA) | 2005 |
| [DQ237900.1](http://www.ncbi.nlm.nih.gov/nuccore/DQ237900.1) | REV-A | Unknown | *pol-env* | TX, USA | 2007 |
| [DQ237901.1](http://www.ncbi.nlm.nih.gov/nuccore/DQ237901.1) | REV-PC-R92 | Attwater prairie chickens | *pol-env* | GA, USA | 2004 |
| [DQ387450](http://www.ncbi.nlm.nih.gov/nuccore/DQ387450).1 | REV | Attwater prairie chickens | Full genome | TX, USA | 2007 |
| [DQ513316.1](http://www.ncbi.nlm.nih.gov/nuccore/DQ513316.1) | REV 3122/03 | Chicken | *env* | Taipei, Taiwan | 2006 |
| [DQ513317.1](http://www.ncbi.nlm.nih.gov/nuccore/DQ513317.1) | REV 3295/04 | Chicken | *env* | Taipei, Taiwan | 2007 |
| [EF526153.1](http://www.ncbi.nlm.nih.gov/nuccore/EF526153.1) | GHV2 strain RM-1 | Lab strain | LTR | GA, USA | 2007 |
| [EF526166](http://www.ncbi.nlm.nih.gov/nuccore/EF526166).1 | GHV2 strain RM-1 | Lab strain | LTR | GA, USA | 2007 |
| [FJ439119](http://www.ncbi.nlm.nih.gov/nuccore/FJ439119).1 | REV 3410/06 | Goose | Full genome | Taipei, Taiwan | 2006 |
| [FJ439120.1](http://www.ncbi.nlm.nih.gov/nuccore/FJ439120.1) | REV /3337/05 | Chicken | Full genome | Taipei, Taiwan | 2005 |
| [FJ496333.1](http://www.ncbi.nlm.nih.gov/nuccore/FJ496333.1) | REV ZD0708 | Unknown | Full genome | China | 2009 |
| [GQ415643](http://www.ncbi.nlm.nih.gov/nuccore/GQ415643).1 | REV HLJR0903 | Chicken | *env* | Heilongjiang, China | 2009 |
| [GQ415644](http://www.ncbi.nlm.nih.gov/nuccore/GQ415644).1 | REV JLR0801 | Chicken | *env* | Heilongjiang, China | 2008 |
| [GQ415645](http://www.ncbi.nlm.nih.gov/nuccore/GQ415645).1 | REV JLR0902 | Chicken | *env* | Heilongjiang, China | 2009 |
| [GQ415646](http://www.ncbi.nlm.nih.gov/nuccore/GQ415646).2 | REV HLJR0901 | Chicken | Full genome | Heilongjiang, China | 2009 |
| [GQ415647](http://www.ncbi.nlm.nih.gov/nuccore/GQ415647).1 | REV JSRD0701 | Duck | *env* | Heilongjiang, China | 2007 |
| [GQ870289.1](http://www.ncbi.nlm.nih.gov/nuccore/GQ870289.1) | GHV2-REV strain GX-0101 | Chicken | LTR | Shandong, China | 2010 |
| [GQ870290.1](http://www.ncbi.nlm.nih.gov/nuccore/GQ870290.1) | FWPV-REV | Chicken | LTR | Shandong, China | 2010 |
| [GU012638.1](http://www.ncbi.nlm.nih.gov/nuccore/GU012638.1) | REV HLJR0902 | Chicken | *env* | Heilongjiang, China | 2009 |
| [GU012639.1](http://www.ncbi.nlm.nih.gov/nuccore/GU012639) | REV HLJR0904 | Chicken | *env* | Heilongjiang, China | 2009 |
| [GU012640.1](http://www.ncbi.nlm.nih.gov/nuccore/GU012640.1) | REV HLJR0801 | Chicken | *env* | Heilongjiang, China | 2008 |
| [GU012641.1](http://www.ncbi.nlm.nih.gov/nuccore/GU012641.1) | REV LNR0801 | Chicken | *env* | Heilongjiang, China | 2008 |
| [GU012642.1](http://www.ncbi.nlm.nih.gov/nuccore/GU012642.1) | REV LNR0802 | Chicken | *env* | Heilongjiang, China | 2008 |
| [GU012643](http://www.ncbi.nlm.nih.gov/nuccore/GU012643).1 | REV HLJR0905 | Chicken | *env* | Heilongjiang, China | 2009 |
| [GU012644](http://www.ncbi.nlm.nih.gov/nuccore/GU012644).1 | REV JLR0803 | Chicken | *env* | Heilongjiang, China | 2008 |
| [GU012645](http://www.ncbi.nlm.nih.gov/nuccore/GU012645).1 | REV JLR0903 | Chicken | *env* | Heilongjiang, China | 2009 |
| [GU012646](http://www.ncbi.nlm.nih.gov/nuccore/GU012646).1 | REV JLR0901 | Chicken | *env* | Heilongjiang, China | 2009 |
| [GU222415](http://www.ncbi.nlm.nih.gov/nuccore/GU222415).1 | Chick syncytial virus (CSV) | Chicken | *env* | USA | 1969 |
| [GU222416.1](http://www.ncbi.nlm.nih.gov/nuccore/GU222416) | REV | Greater prairie chicken | *env* | USA | 1996 |
| [GU222417.1](http://www.ncbi.nlm.nih.gov/nuccore/GU222417) | REV | Chicken | *env* | USA | 1993 |
| [GU222418.1](http://www.ncbi.nlm.nih.gov/nuccore/GU222418.1) | REV | Turkey | *env* | USA | 2000 |
| [GU222419.1](http://www.ncbi.nlm.nih.gov/nuccore/GU222419.1) | REV | Turkey | *env* | USA | 1958 |
| [GU222420](http://www.ncbi.nlm.nih.gov/nuccore/GU222420).1 | REV | Turkey | *env* | USA | 1974 |
| [GU969140.1](http://www.ncbi.nlm.nih.gov/nuccore/GU969140.1) | REV JLR0802 | Chicken | *env* | Heilongjiang, China | 2008 |
| [HQ111429.1](http://www.ncbi.nlm.nih.gov/nuccore/HQ111429.1) | FWPV-REV strain DCEP | Vaccine | LTR | China | 2010 |
| [M22224.1](http://www.ncbi.nlm.nih.gov/nuccore/M22224.1) | REV | Lab strain | LTR | USA | 1987 |
| [M87666.1](http://www.ncbi.nlm.nih.gov/nuccore/M87666.1) | SNV | Lab strain | *env* | USA | 1992 |
| [S70398.1](http://www.ncbi.nlm.nih.gov/nuccore/S70398.1) | pREVA6 plasmid | Chicken | LTR | USA | 1994 |
| [S79845.1](http://www.ncbi.nlm.nih.gov/nuccore/S79845.1) | GHV2-REV strain JM-Hi3 | Chicken | LTR | USA | 1992 |
| [S82226.1](http://www.ncbi.nlm.nih.gov/nuccore/S82226.1) | GHV2-REV strain RM-1 | Lab strain | LTR | USA | 1997 |
| [X01455.1](http://www.ncbi.nlm.nih.gov/nuccore/X01455.1) | REV-A | Lab strain | *pol-env* | USA | 1984 |
