# Supplementary material for: The Extraordinary Evolutionary History of the Reticuloendotheliosis Viruses
Source: PLoS Biol. 2013 Aug 27;11(8):e1001642. doi: 10.1371/journal.pbio.1001642 (PMC3754887; doi:10.1371/journal.pbio.1001642)
Supplement: Table S5 — aState or prefecture/two-letter ISO country code. (DOC) [file pbio.1001642.s007.doc]

**Table S5: REV serology**

| **Year** | **Strain** | **Origin** | **Animal status** | **Location** ^a^ | **Reference** |
| --- | --- | --- | --- | --- | --- |
| 1969 | REV | Chickens | Domestic | US | [1] |
| 1977 | REV | Chickens | Domestic | JP | [2] |
| 1981 | REV | Chickens | Domestic | DE | [3] |
| 1982 | REV | Chickens | Domestic | NZ | [4] |
| 1982 | REV | Chickens | Domestic | US | [5] |
| 1982 | REV | Chickens | Domestic | PG | [6] |
| 1984 | REV | Turkey | Domestic | US | [7] |
| 1985 | REV | Chickens | Domestic | MO, US | [8] |
| 1993 | REV | Turkey, chicken | Domestic | NG | [9] |
| 1993 | REV | Chickens | Domestic | IL | [10] |
| 1993 | REV | Chickens, ducks, pheasants | Domestic | JP | [11] |
| 1994 | REV | Ostriches | Domestic | ZW | [12] |
| 1994 | REV | Chickens | Domestic | ZW | [13] |
| 2001 | REV | Turkey | Domestic | DE | [14] |
| 2002 | REV | Turkeys, lesser prairie chickens | Wild | TX, US | [15, 16] |
| 2008 | REV | REV | Domestic and experimental | BR | [17] |
| 2009 | REV | Prairie chickens | Domestic | US | [18] |
|  |  |  |  |  |  |

**References**

1. Aulisio, C.G. and A. Shelokov, *Prevalence of reticuloendotheliosis in chickens: immunofluorescence studies.* Proceedings of the Society for Experimental Biology and Medicine. Society for Experimental Biology and Medicine, 1969. **130**(1): p. 178-81.

2. Wakabayashi, T. and H. Kawamura, *Serological survey of reticuloendotheliosis virus infection among chickens in Japan.* National Institute of Animal Health quarterly, 1977. **17**(2): p. 73-4.

3. Neumann, U., et al., *[Serological survey on the status of infection with reticuloendotheliosis virus in Northern Germany with regard to epidemiological aspects (author's transl)].* DTW. Deutsche tierarztliche Wochenschrift, 1981. **88**(3): p. 104-7.

4. Howell, L.J., T.J. Bagust, and A.M. Alexander, *Serological investigations of infectious bursal disease virus and reticuloendotheliosis virus infections in New Zealand chickens.* New Zealand veterinary journal, 1982. **30**(8): p. 128.

5. Witter, R.L., et al., *Serologic evidence in commercial chicken and turkey flocks of infection with reticuloendotheliosis virus.* Avian diseases, 1982. **26**(4): p. 753-62.

6. Van Kammen, A., *Survey of some poultry viruses in Papua New Guinea.* Tropical animal health and production, 1982. **14**(2): p. 109-19.

7. Witter, R.L. and S.E. Glass, *Reticuloendotheliosis in breeder turkeys.* Avian diseases, 1984. **28**(3): p. 742-50.

8. Witter, R.L. and D.C. Johnson, *Epidemiology of reticuloendotheliosis virus in broiler breeder flocks.* Avian diseases, 1985. **29**(4): p. 1140-54.

9. Okoye, J.O., W. Ezema, and J.N. Agoha, *Naturally occurring clinical reticuloendotheliosis in turkeys and chickens.* Avian pathology : journal of the W.V.P.A, 1993. **22**(2): p. 237-44.

10. Meroz, M., *Reticuloendotheliosis and 'pullet disease' in Israel.* The Veterinary record, 1992. **130**(5): p. 107-8.

11. Sasaki, T., S. Sasaki, and H. Koyama, *A survey of an antibody to reticuloendotheliosis virus in sera of chickens and other avian species in Japan.* The Journal of veterinary medical science / the Japanese Society of Veterinary Science, 1993. **55**(5): p. 885-8.

12. Cadman, H.F., et al., *A serosurvey using enzyme-linked immunosorbent assay for antibodies against poultry pathogens in ostriches (Struthio camelus) from Zimbabwe.* Avian diseases, 1994. **38**(3): p. 621-5.

13. Kelly, P.J., et al., *Diseases and management of backyard chicken flocks in Chitungwiza, Zimbabwe.* Avian diseases, 1994. **38**(3): p. 626-9.

14. Hafez, H.M., *Serological investigations on reticuloendotheliosis in turkey flocks.* Journal of veterinary medicine. B, Infectious diseases and veterinary public health, 2001. **48**(7): p. 547-50.

15. Peterson, M.J., et al., *Infectious disease survey of Rio Grande wild turkeys in the Edwards Plateau of Texas.* J Wildl Dis, 2002. **38**(4): p. 826-33.

16. Peterson, M.J., et al., *Infectious disease survey of lesser prairie chickens in north Texas.* Journal of wildlife diseases, 2002. **38**(4): p. 834-9.

17. Santos, V.L., et al., *Detection of reticuloendotheliosis virus by immunohistochemistry and in situ hybridization in experimentally infected Japanese quail embryos and archived formalin-fixed and paraffin-embedded tumours.* Avian pathology : journal of the W.V.P.A, 2008. **37**(4): p. 451-6.

18. Drechsler, Y., et al., *An avian, oncogenic retrovirus replicates in vivo in more than 50% of CD4+ and CD8+ T lymphocytes from an endangered grouse.* Virology, 2009. **386**(2): p. 380-6.
