# Supplementary material for: The Extraordinary Evolutionary History of the Reticuloendotheliosis Viruses
Source: PLoS Biol. 2013 Aug 27;11(8):e1001642. doi: 10.1371/journal.pbio.1001642 (PMC3754887; doi:10.1371/journal.pbio.1001642)
Supplement: Table S6 — Asterisks indicate studies sponsored by the US Office of Scientific Research and Development (OSRD). (DOC) [file pbio.1001642.s008.doc]

**Table S6. Experimental *Plasmodium lophurae* infections, 1938-1946.**

| **Date** | **Location** | **Species infected** | **Reference** |
| --- | --- | --- | --- |
|  |  |  |  |
| 1938 | Princeton, NJ | Chickens | [1] |
| 1940 | Chicago, IL | Chickens | [2] |
| 1941 | Tennessee Valley, AL | *Anopheles quadrilmaculatus* | [3] |
| 1941 | Baltimore, MD | Duck/Anopheles | [4] |
| 1941 | Baltimore, MD | Chickens | [5, 6] |
| 1941 | Princeton, NJ | Ducks | [7] |
| 1941 | Princeton, NJ | Ducks | [8] |
| 1941 | New Brunswick, NJ | Ducks | [9] |
| 1942 | Memphis TN | Ducks | [10, 11] |
| 1942 | Tennessee Valley, AL | *Anopheles quadrilmaculatus* | [12] |
| 1943 | Memphis TN | Ducks | [13] |
| 1943 | Rahway, NJ | Ducks | [14] |
| 1943 | Princeton, NJ | Chickens & ducks | [15, 16] |
| 1944 | Little Rock, AK | Ducks | [17] |
| 1944 | Baltimore, MD | Chickens & ducks | [18] |
| 1944 | Rahway, NJ | Chickens | [19-21] |
| 1944 | Baltimore, MD | Turkeys | Communication by Porter & Laird, referenced in [22] |
| 1945 | Baltimore MD | Ducks | [23]* |
| 1945 | New York, NY | Ducks | [24] |
| 1945 | Chicago, IL | Ducks | [25] |
| 1945 | Memphis TN | Chickens | [26] |
| 1945 | Chicago, IL | Chickens | [27, 28]* |
| 1945 | London, UK | Turkeys | [22] |
| 1945 | Baltimore, MD | Ducks | [29] |
| 1945 | Chicago, IL | Chickens | [30]* |
| 1946 | Baltimore, MD | Ducks | [31]* |
| 1946 | Baltimore, MD | Ducks | [32, 33]* |
| 1946 | Baltimore, MD | Ducks | [34]* |
| 1946 | Chicago, IL | Ducks | [35]* |
| 1946 | Little Rock, AK | Ducks | [36, 37] |
| 1946 | Boston, MA | Ducks | [38] |
| 1946 | Little Rock, AK | Ducks | [39] |
| 1947 | Chicago, IL | Ducks/Chickens | [40]* |
| 1947 | Little Rock, AK | Ducks | [41-44] |
| 1947 | London, UK | Cell culture | [45] |
| 1947 | Princeton, NJ | Ducks | [46-48] |
|  |  |  |  |

**References**

1. Coggeshall, L.T., *Plasmodium lophurae, a new species of malaria pathogenic for the domestic fowl.* Am. J. Hyg. , 1938. **27**: p. 615–618.

2. Taliaferro, W.H. and L.G. Taliaferro, *Active and passive immunity in chickens against Plasmodium lophurae.* Journal of Infectious Diseases, 1940. **66**: p. 153-165.

3. Hurlbut, H.S. and R. Hewitt, *Sporozoites of Plasmodium Lophurae, an Avian Malaria Parasite, in Anopheles Quadrimaculatus.* Public Health Reports, 1941. **56**(26): p. 1336-1337.

4. Laird, R.L., *Observations on mosquito transmission of Plasmodium lophurae.* American journal of hygiene, 1941. **34**(1/3): p. 163-167.

5. Terzian, L.A., *Studies on Plasmodium lophurae, a malarial parasite in fowls.* American Journal of Epidemiology, 1941. **33**(1): p. 1-22.

6. Terzian, L.A., *Studies on Plasmodium lophurae, a malarial parasite in fowls I Biological characteristics.* American journal of hygiene, 1941. **33**(1/3): p. 1-22.

7. Trager, W., *STUDIES ON CONDITIONS AFFECTING THE SURVIVAL IN VITRO OF A MALARIAL PARASITE (PLASMODIUM LOPHURAE).* J Exp Med, 1941. **74**(5): p. 441-62.

8. Trager, W., *The effect of intraperitoneal injections of carbon ink on the course of Plasmodium lophurae infections in chickens.* American journal of hygiene, 1941. **34**(1/3): p. 141-149.

9. Walker, H.A. and H.B. van Dyke, *Control of malaria infection (P lophurae) in ducks by sulfonamides.* Proceedings of the Society for Experimental Biology and Medicine, 1941. **48**(1): p. 368-372.

10. Hewitt, R., *Studies on the host-parasite relationships of untreated infections with Plasmodium lophurae in ducks.* American journal of hygiene, 1942. **36**(1): p. 6-U6.

11. Hewitt, R.I., A.P. Richardson, and L.D. Seager, *Observations of untreated infections with Plasmodium lophurae in twelve hundred young white Pekin ducks.* American Journal of Epidemiology, 1942. **36**(3): p. 362-373.

12. Hurlbut, H.S. and R. Hewitt, *The Transmission of Plasmodium Lophurae, an Avian Malaria Parasite, by Anopheles Quadrimaculatus.* Public Health Reports, 1942. **57**(50): p. 1891-1892.

13. Hewitt, R.I. and A.P. Richardson, *The direct plasmodicidal effect of quinne, atabrine and plasmochin on Plasmodium lophurae.* Journal of Infectious Diseases, 1943. **73**: p. 1-11.

14. Seeler, A.O., E. Dusenbery, and C. Malanga, *The comparative activity of quinine, quinidine, cinchonine, cinchonidine and quinoidine against Plasmodium lophurae infections of Pekin ducklings.* Journal of Pharmacology and Experimental Therapeutics, 1943. **78**(2): p. 159-163.

15. Trager, W., *FURTHER STUDIES ON THE SURVIVAL AND DEVELOPMENT IN VITRO OF A MALARIAL PARASITE.* J Exp Med, 1943. **77**(5): p. 411-20.

16. Trager, W., *THE INFLUENCE OF BIOTIN UPON SUSCEPTIBILITY TO MALARIA.* J Exp Med, 1943. **77**(6): p. 557-82.

17. Rigdon, R.H., *A pathological study of the acute lesions produced by Plasmodium lophurae in young white Pekin ducks.* American Journal of Tropical Medicine, 1944. **24**: p. 371-377.

18. Jeffery, G.M., *Investigations on the mosquito transmission of Plasmodium lophurae coggeshall, 1938.* American journal of hygiene, 1944. **40**(3): p. 251-263.

19. Seeler, A.O., *Effect of pyridoxine on activity of quinine and atabrine against Plasmodium lophurae infections.* Proceedings of the Society for Experimental Biology and Medicine, 1944. **57**(1): p. 113-115.

20. Seeler, A.O. and W.H. Ott, *Effect of riboflavin deficiency on the course of Plasmodium lophurae infection in chicks.* Journal of Infectious Diseases, 1944. **75**: p. 175-178.

21. Seeler, A.O., W.H. Ott, and M.E. Gundel, *Effect of biotin deficiency on the course of Plasmodium lophurae infection in chicks.* Proceedings of the Society for Experimental Biology and Medicine, 1944. **55**(2): p. 107-109.

22. Tonkin, I.M., *Exoerythrocytic forms of Plasmodium lophurae in turkeys.* Transactions of the Royal Society of Tropical Medicine and Hygiene, 1945. **39**(4): p. 271.

23. Bratton, A.C., Jr., *Continuous intravenous chemotherapy of Plasmodium lophurae infection in ducks.* J Pharmacol Exp Ther, 1945. **85**: p. 103-10.

24. Freund, J., H.E. Sommer, and A.W. Walter, *Immunization against Malaria: Vaccination of Ducks with Killed Parasites Incorporated with Adjuvants.* Science, 1945. **102**(2643): p. 200-2.

25. Jacobs, H.R., *Immunization against Malaria - Unsuccessful Attempts to Increase Resistance of Ducklings to Plasmodium Lophurae Infections by Previous Injections of Materials Containing the Forssman Antigen.* American Journal of Tropical Medicine, 1945. **25**(2): p. 151-153.

26. Rigdon, R.H., *Plasmodium-Lophurae Infection of the Chick Embryo.* American journal of hygiene, 1945. **42**(2): p. 189-194.

27. Taliaferro, W.H. and L.G. Taliaferro, *Immunological relationships of Plasmodium gallinaceum and Plasmodium lophurae.* The Journal of infectious diseases, 1945. **77**: p. 224-48.

28. Taliaferro, W.H., L.G. Taliaferro, and E.L. Simmons, *Increased parasitemia in chicken malaria (Plasmodium gallinaceum and Plasmodium lophurae) following x-irradiation.* The Journal of infectious diseases, 1945. **77**: p. 158-76.

29. Wolfson, F., *An Experimental Study of Mixed Infections with Plasmodium-Cathemerium and Plasmodium-Lophurae in Ducks.* American journal of hygiene, 1945. **41**(1): p. 123-135.

30. Zuckerman, A., *Invitro Opsonic Tests with Plasmodium-Gallinaceum and Plasmodium-Lophurae.* Journal of Infectious Diseases, 1945. **77**(1): p. 28-59.

31. Bovarnick, M.R., A. Lindsay, and L. Hellerman, *Preparation and properties of Plasmodium lophurae separated from the red cells of duck blood by means of saponin.* The Journal of biological chemistry, 1946. **163**: p. 523-33.

32. Dearborn, E.H., *Filtrable agents lethal for ducks.* Proc Soc Exp Biol Med, 1946. **63**(1): p. 48.

33. Dearborn, E.H. and E.K. Marshall, Jr., *Curative action of drugs in lophurae malaria of the duck.* Proceedings of the Society for Experimental Biology and Medicine. Society for Experimental Biology and Medicine, 1946. **63**(1): p. 46-8.

34. Hellerman, L., M.R. Bovarnick, and C.C. Porter, *Metabolism of the Malarial Parasite - Action of Antimalarial Agents Upon Separated Plasmodium-Lophurae and Upon Certain Isolated Enzyme Systems.* Federation proceedings, 1946. **5**(3): p. 400-405.

35. Kelsey, F.E., F.K. Oldham, and A.L. Gittelson, *Curative effect of plasmoquin in Plasmodium lophurae infections.* Federation proceedings, 1946. **5**(1 Pt 2): p. 185.

36. Rigdon, R.H., *Effect of vitamin A deficiency on Plasmodium lophurae infection in ducks.* The Journal of infectious diseases, 1946. **79**(3): p. 272-7.

37. Rigdon, R.H. and H.H. Rostorfer, *Effect of oxygen on P. lophurae infected ducks.* Proceedings of the Society for Experimental Biology and Medicine. Society for Experimental Biology and Medicine, 1946. **63**(1): p. 165-7.

38. Roos, A., D.M. Hegsted, and F.J. Stare, *Nutritional studies with the duck; the effect of vitamin deficiencies on the course of P. lophurae infection in the duck and the chick.* The Journal of nutrition, 1946. **32**(5): p. 473-84.

39. Rostorfer, H.H. and G.H. Mc, *Some factors which decrease arterial saturation in bird malaria-ducks infected with P. lophurae.* Proceedings of the Society for Experimental Biology and Medicine. Society for Experimental Biology and Medicine, 1946. **62**(2): p. 151-4.

40. Huff, C.G., F. Coulston, and et al., *Preerythrocytic development of Plasmodium lophurae in various hosts.* The Journal of infectious diseases, 1947. **81**(1): p. 7-13.

41. Rigdon, R.H., *An Invitro Study of the Mechanism Producing Rapid Diminution in the Parasitemia in Ducks Infected with Plasmodium-Lophurae.* American journal of hygiene, 1947. **46**(2): p. 254-259.

42. Rigdon, R.H. and H.N. Marvin, *Effect of Insulin on Plasmodium-Lophurae Infection in Ducks.* American journal of hygiene, 1947. **45**(2): p. 185-190.

43. Rigdon, R.H. and B.E. Mccain, *Some Factors That Influence the Degree of Parasitemia in Ducks Infected with P-Lophurae.* American Journal of Tropical Medicine, 1947. **27**(6): p. 673-681.

44. Rigdon, R.H. and H.H. Rostorfer, *Observations on the anemia in ducks infected with P. lophurae.* Blood, 1947. **2**(3): p. 244-55.

45. Tonkin, I.M. and F. Hawking, *Growth of Protozoa in tissue culture; Plasmodium lophurae, exoerythrocytic forms, in vivo and in vitro.* Transactions of the Royal Society of Tropical Medicine and Hygiene, 1947. **41**(3): p. 407-14.

46. Trager, W., *The resistance of certain adult ducks to infection by the avian malaria parasite Plasmodium lophurae.* The Journal of parasitology, 1947. **33**(2): p. 12.

47. Trager, W., *The Relation to the Course of Avian Malaria of Biotin and a Fat-Soluble Material Having the Biological Activities of Biotin.* The Journal of experimental medicine, 1947. **85**(6): p. 663-83.

48. Trager, W., *The Development of the Malaria Parasite Plasmodium-Lophurae in Red Blood Cell Suspensions Invitro.* Journal of Parasitology, 1947. **33**(4): p. 345-350.
